# Alternative methods for skeletal maturity estimation with the EOS scanner—Experience from 934 patients

**DOI:** 10.1371/journal.pone.0267668

**Published:** 2022-05-06

**Authors:** Ádám Tibor Schlégl, Ian O’Sullivan, Péter Varga, Péter Than, Csaba Vermes

**Affiliations:** 1 Department of Orthopaedics, University of Pécs, Medical School, Pécs, Hungary; 2 Department of Primary Health Care, University of Pécs, Medical School, Pécs, Hungary; University Tunku Abdul Rahman, MALAYSIA

## Abstract

**Background:**

Hand-wrist bone age assessment methods are not possible on typical EOS 2D/3D images without body position modifications that may affect spinal position. We aimed to identify and assess lesser known bone age assessment alternatives that may be applied retrospectively and without the need for extra imaging.

**Materials and methods:**

After review of 2857 articles, nine bone age methods were selected and applied retrospectively in pilot study (thirteen individuals), followed by evaluation of EOS images of 934 4-24-year-olds. Difficulty of assessment and time taken were recorded, and reliability calculated.

**Results:**

Five methods proved promising after pilot study. Risser ‘plus’ could be applied with no difficulty in 89.5% of scans (836/934) followed by the Oxford hip method (78.6%, 734/934), cervical (79.0%, 738/934), calcaneus (70.8%, 669/934) and the knee (68.2%, 667/934). Calcaneus and cervical methods proved to be fastest at 17.7s (95% confidence interval, 16.0s to 19.38s & 26.5s (95% CI, 22.16s to 30.75s), respectively, with Oxford hip the slowest at 82.0 s (95% CI, 76.12 to 87.88s). Difficulties included: regions lying outside of the image—assessment was difficult or impossible in upper cervical vertebrae (46/934 images 4.9%) and calcaneus methods (144/934 images, 15.4%); position: lower step length was associated with difficult lateral knee assessment & head/hand position with cervical evaluation; and resolution: in the higher stages of the hip, calcaneal and knee methods.

**Conclusions:**

Hip, iliac crest and cervical regions can be assessed on the majority of EOS scans and may be useful for retrospective application. Calcaneus evaluation is a simple and rapidly applicable method that may be appropriate if consideration is given to include full imaging of the foot.

## Introduction

Skeletal maturity is of interest to the paediatric orthopaedist, endocrinologist, paediatrician, and orthodontist, as well as forensic physicians or radiologist. As the skeletal maturity can be a reliable indicator of the biological age, it is used in diagnosis, in timing of treatment (e.g. scoliosis, leg length discrepancy, orthodontia etc.) or in age estimation [1].

**Fig 1:**
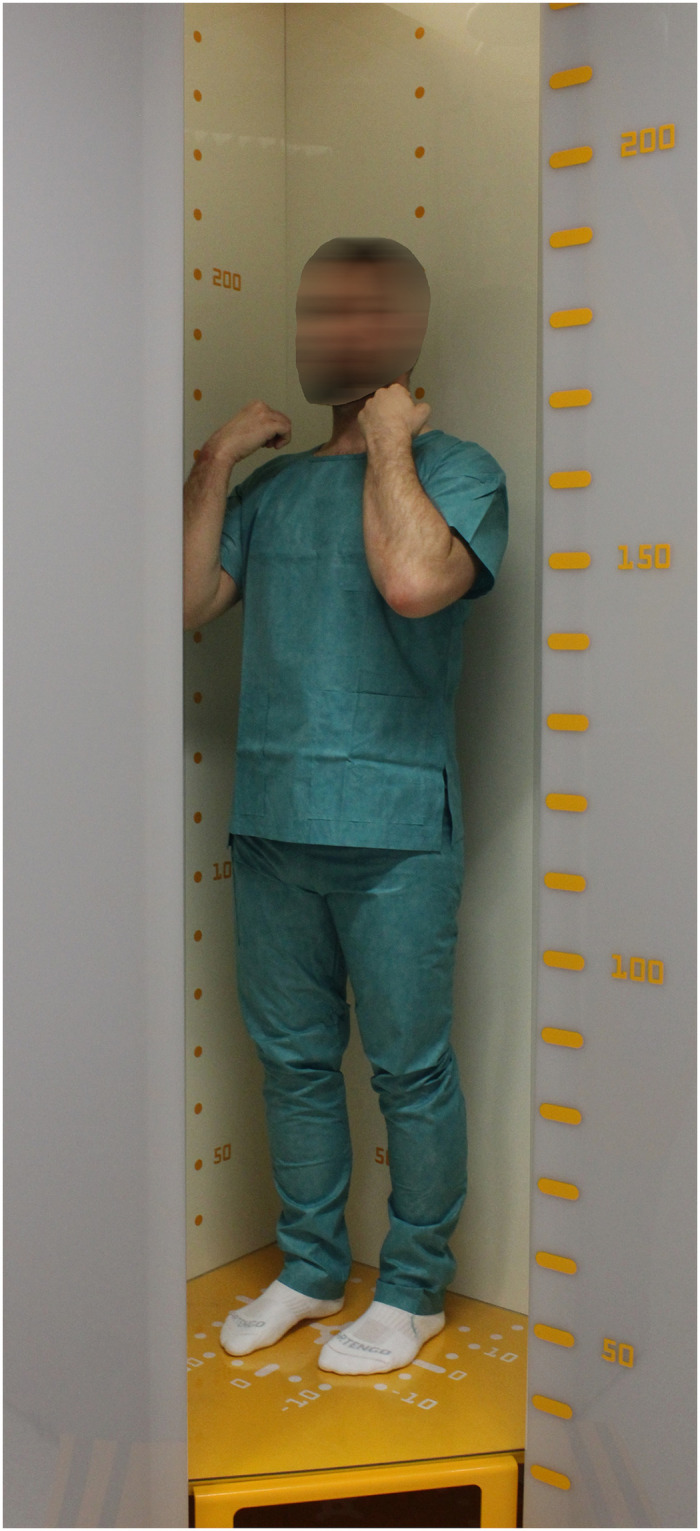
Patient position inside the EOS 2D/3D scanner.

While the EOS scanner has recently gained popularity in the assessment of scoliosis due to its low radiation dose [2, 3], the position required for spinal imaging does not permit evaluation of the hand or wrist (see Fig 1), the region favoured by more than 97.6% of US paediatric radiologists for assessing 3–18 year-olds [4]. While the Risser system can be applied, the first stage typically occurs after onset of peak height velocity and as such is only useful for predicting the end of the risk period for curve progression [5, 6].

In the present study we aimed to identify and present promising alternative bone age methods that may be of use to the clinician working with the EOS, and to evaluate their reliability and usability.

## Materials and methods

### Literature review

A Pubmed search was conducted on March 30 2016 using terms “bone age”, “skeletal age” and “skeletal maturation”, and 185 different methods were identified (see S1 Table for a comprehensive list). Nine promising methods were selected:

1. Calcaneus [7];
2. Cervical vertebrae [8];
3. Clavicle [9];
4. Shoulder [10];
5. Elbow [11];
6. First rib [12];
7. Oxford Hip [13];
8. Iliac apophysis and tri-radiate cartilage: Risser ‘plus’ method [14];
9. Knee [15].

After discussion, clavicle, rib, and elbow methods were not included due to insufficient resolution (clavicle) or severe shadowing of landmarks due to patient position in the EOS (first rib, elbow).

### Pilot study

Three graders (one orthopaedic resident and two PhD candidate medical doctors) were given text and pictorial descriptions of the remaining methods and trained with assistance of a senior orthopaedic specialist and a senior radiologist. 13 normal children aged 3–16 were randomly selected from our database of EOS scans taken during 2007–2016 and images evaluated by each method three times by the three observers, on separate days.

### Method testing

**Fig 2:**
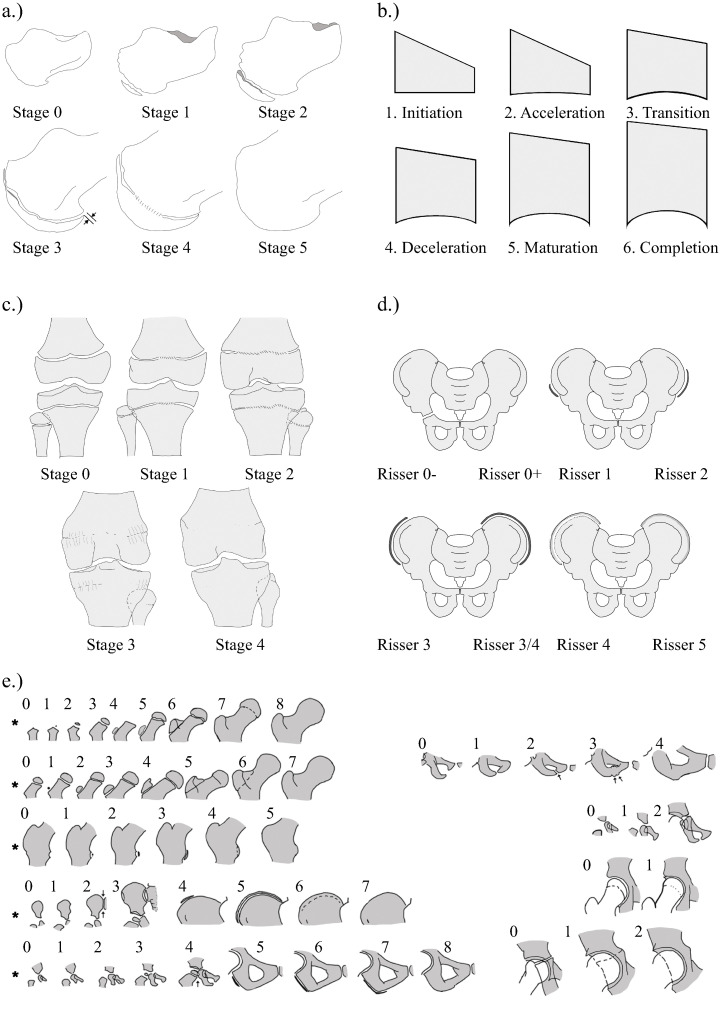
(a-e). Pictorial illustration of five bone age estimation methods. (a) Calcaneus, (b) Cervical, (c) Knee, (d) Risser Plus and (e) Oxford Hip methods. See S1 Fig for more in depth description of each method. (Reproduced from O’Sullivan et al. [17], creative commons license https://creativecommons.org/licenses/by/4.0/legalcode).

After pilot study five methods (see Fig 2a–2e) were assessed based on:

1. Reliability: 30 images were randomly selected and assessed three times by each of the three observers, on three separate days, and intraclass correlation coefficient (ICC) estimated.
2. Difficulty of Assessment: methods were assessed based on a four-point Likert scale: ‘1’ easy—method was easy to apply; ‘2’ moderate—some minor exposure problems or minor obstruction, but evaluation could confidently be made; ‘3’ difficult—significant obstruction, image partially cut eg. 1/3 or less of a landmark obscured or not visible, such that assumption must be made; ‘4’ impossible–landmark not in image or totally obstructed. In the hip and knee methods, if the sum of problematic landmarks exceeded 2 or 3, respectively, then the whole image was regarded as ‘difficult’.
3. Speed: Two observers used digital timers to record evaluation time with each method during their final 200 randomised images.

EOS images of disease-free children and adolescents were retrospectively collected from our database taken during normal clinical practice from 2007–2016, a total of 7108 full body image pairs. Selection criteria were: individuals aged 4–24 years old; absence of any disorder or previous surgery affecting skeletal anatomy; absence of movement artefacts. Individuals from age group 17–24 were limited to 50 per year (25 males and 25 females). 59 images were damaged or missing from our database resulting finally in 934 disorder-free individuals. Image-pairs were randomised and assigned equally to the three graders. All scans were performed with orthopaedic indication (joint pain with unknown origin, suspicion of scoliosis or functional kyphosis) but upon imaging, no deformity was revealed.

For randomisation and selection, Microsoft Excel v14.0.6112.5000 (Microsoft Corp., Redmond, WA) software was used. Informed written consent at the time of imaging was attained from all individuals, or their guardians. Institutional Review Board ethical permission was granted for this study and all work was in accordance with the Declaration of Helsinki (Institutional and Regional Scientific Ethical Committee of University of Pécs, permission No: 7607-PTE2019).

For a deeper description of methods applied, see S1 Fig.

## Results

### Literature review

Online search yielded 4758 articles: “bone age” returned 3230 results, “skeletal age”: 808, “and “skeletal maturation”: 1153. Duplicate (433) or irrelevant articles (516) and publications in which the method was unlisted (555), absent from the abstract (foreign language articles) (500) or the article could not be located (330) were removed, leaving 2857 articles.

### Pilot study

After pilot study, the shoulder method was no longer included as observers found serious difficulties evaluating the region in 54–72% of scans. Only one of the three required landmarks were found to be assessable in 23–38% of scans (3–5 of 13 scans), the apex/ angle of the coracoid process was not visible in 15–38% (2–5 of 13 scans) in addition to low satisfaction reported by observers using the method.

Plantar sesamoid identification is recommended to assist with the calcaneal method, however sesamoids could not be clearly identified in any scans, and a ‘possible’ presence reported in four of 13 scans. Identification difficulties were partly due to the absence of the dorso-volar plane in EOS images but also due to deterioration of image resolution at the inferior image edge. In one incidence a patient with conventional X-ray of the foot taken at the same time received a negative report on the presence of sesamoids after EOS review, despite their clear presence with conventional image.

All other methods could be assessed satisfactorily.

### Primary study

**Fig 3:**
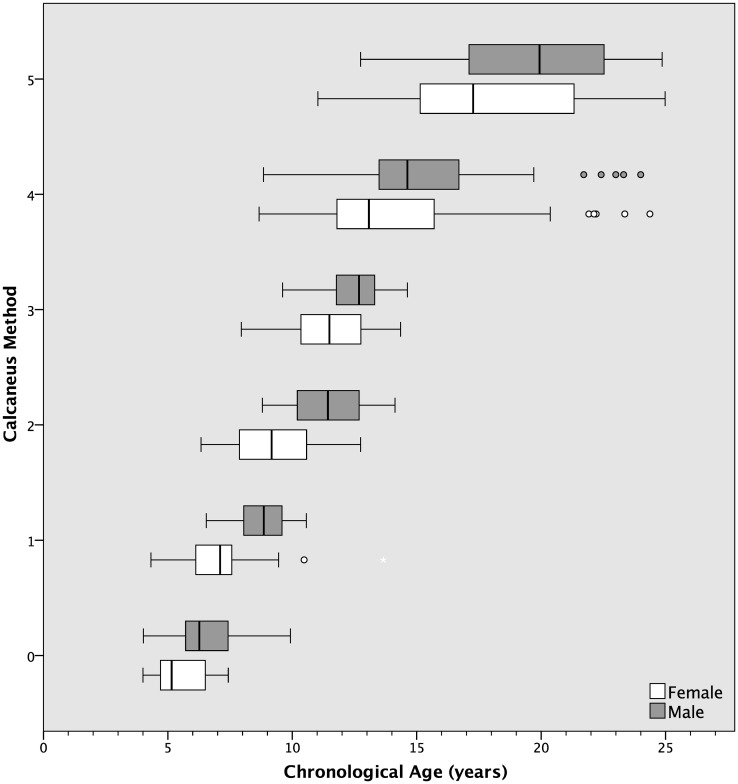
Distribution of chronological ages at each bone age stage with calcaneus method. Gender is shown at each stage.

**Fig 4:**
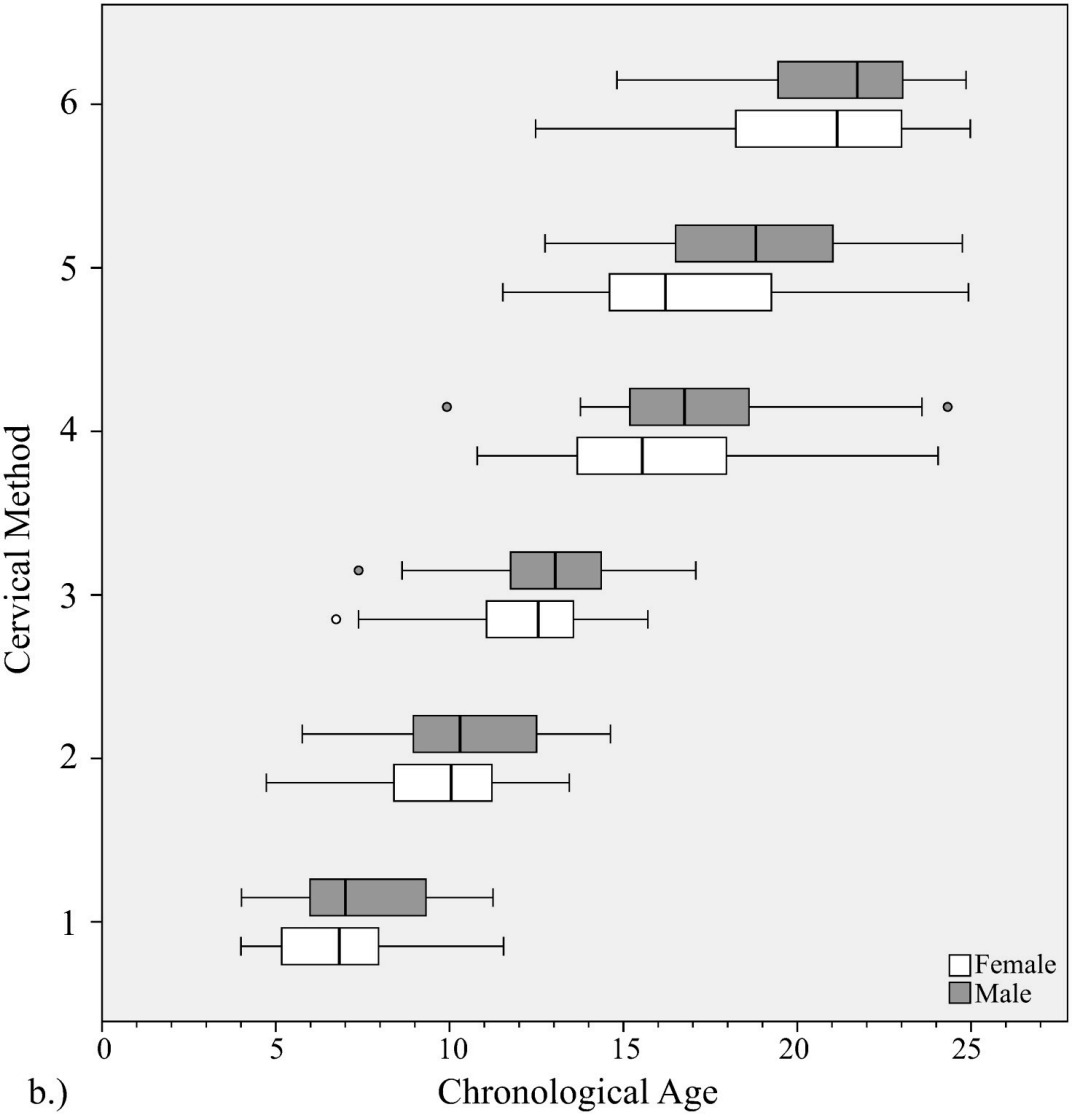
Distribution of chronological ages at each bone age stage with cervical method. Gender is shown at each stage.

**Fig 5:**
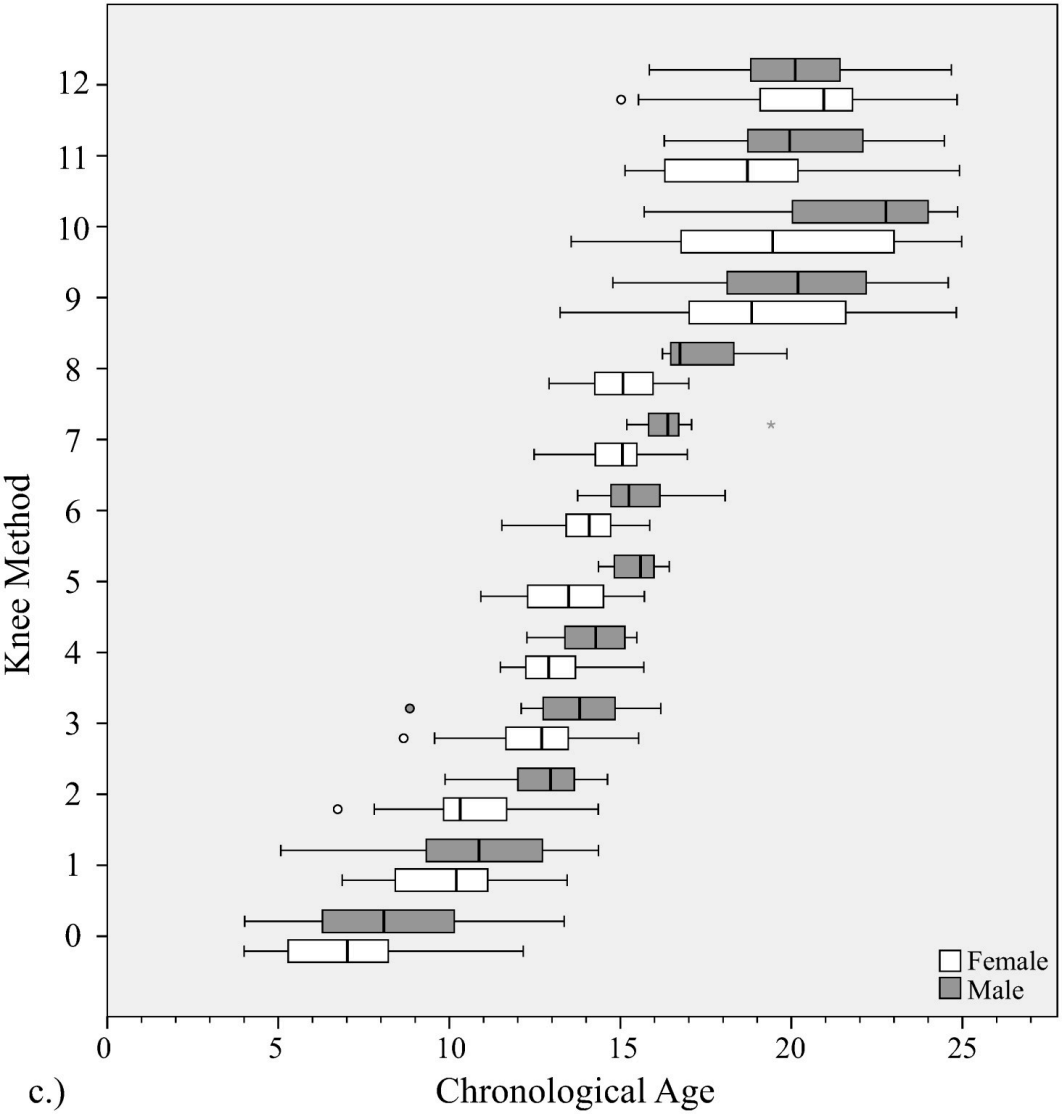
Distribution of chronological ages at each bone age stage with knee method. Gender is shown at each stage.

**Fig 6:**
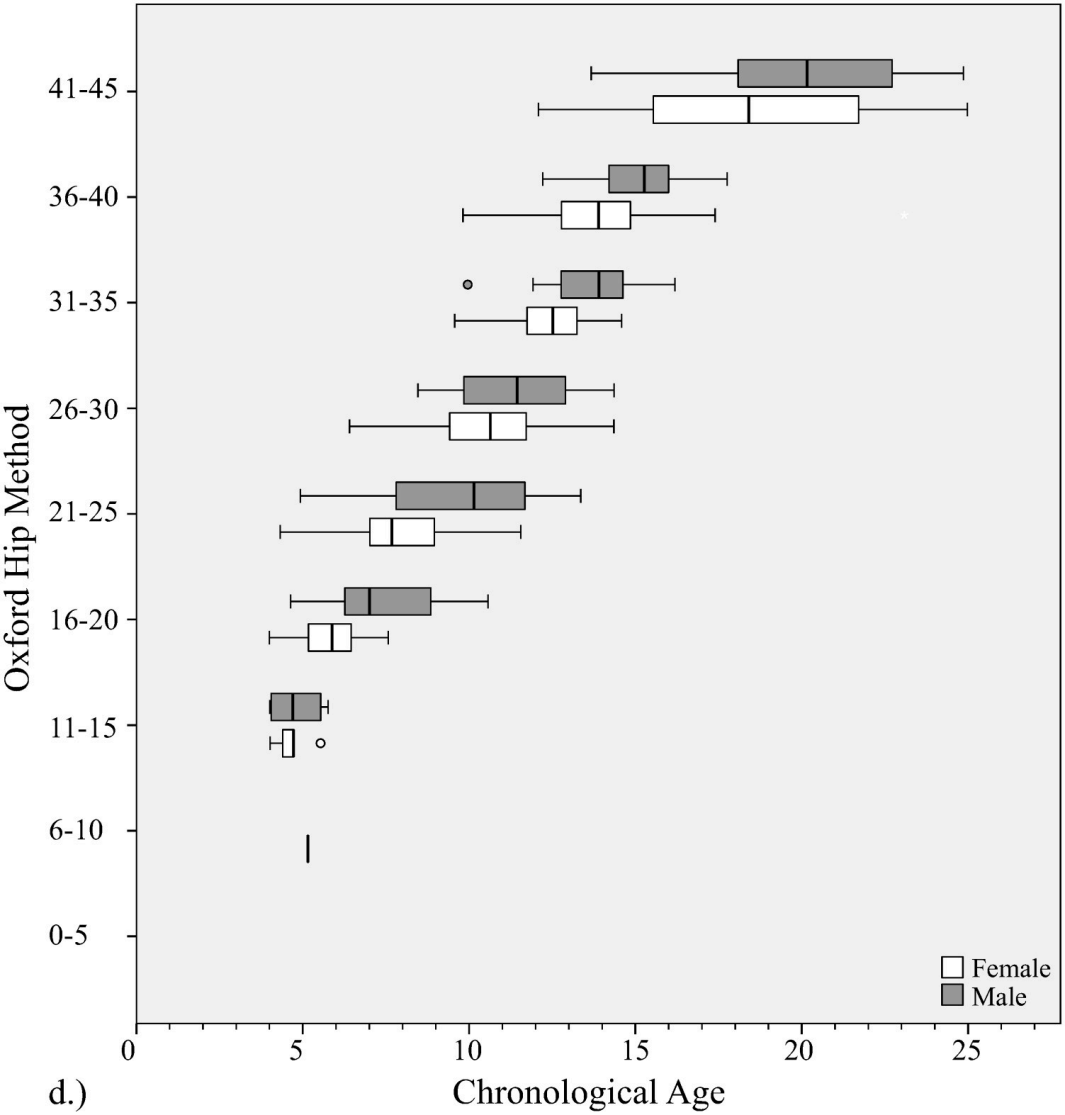
Distribution of chronological ages at each bone age stage with Oxford hip method. Gender is shown at each stage.

**Fig 7:**
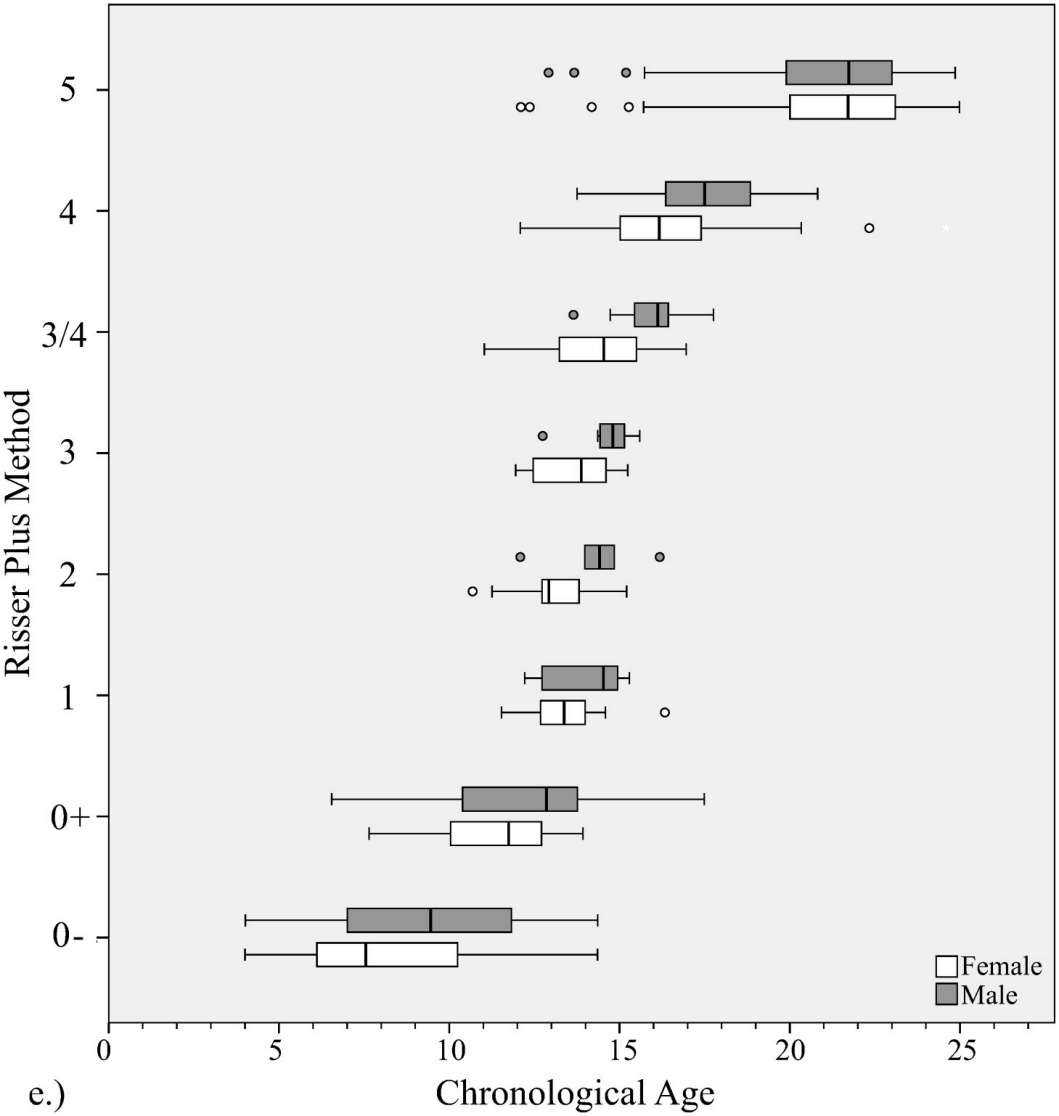
Distribution of chronological ages at each bone age stage with Risser plus method. Gender is shown at each stage.

Bone ages of individuals with each method are shown in Figs 3–7.

#### 1. Reliability

We previously reported excellent inter- and intra-observer reliability values using the cervical vertebral method [16], and excellent values were also found with calcaneus, Risser ‘plus’ system and Oxford hip methods [17]. The knee method, although ‘good’, was not as reliable. ICC values are shown in Table 1.

**Table 1:** Reliability.

	Calcaneus	Cervical	Knee	Oxford Hip	Risser Plus
**Inter-observer reliability**	**0.945**	**0.976**	0.865	**0.902**	**0.94**
**Intra-observer reliability**	**0.953–0.999**	**0.949–0.959**	0.841–**0.956**	**0.949–0.993**	**0.982–0.969**

Inter- and intra-observer reliability assessed by Intraclass Correlation Coefficient calculation. Those with ICC >0.9 were regarded as ‘excellent’ and marked with bold script (ICC = intraclass correlation coefficient). Minimum and maximum intra-observer reliability values are shown only. (Reproduced from O’Sullivan et al. [17], creative commons license https://creativecommons.org/licenses/by/4.0/legalcode).

#### 2. Difficulty of assessment

**Fig 8:**
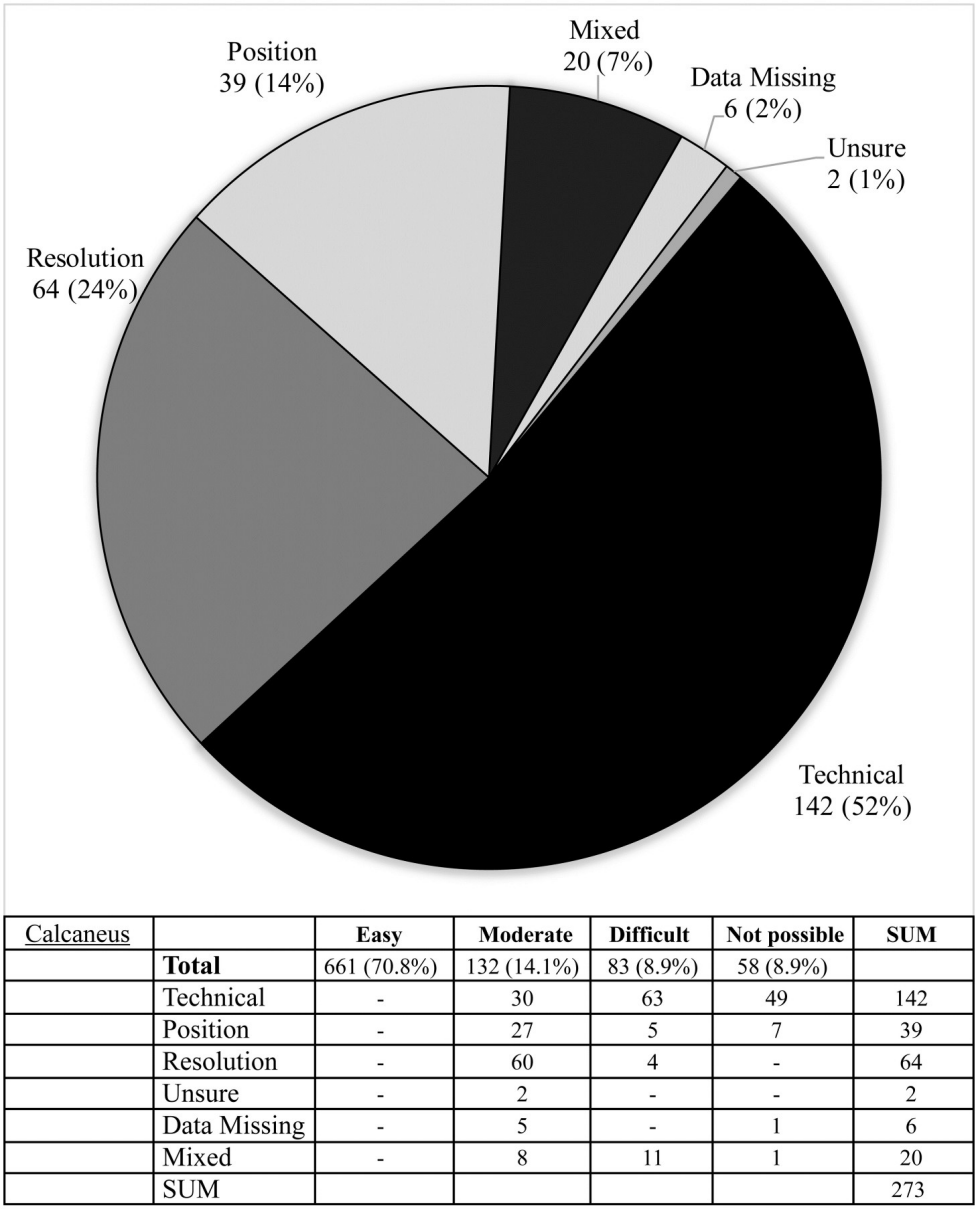
Difficulties reported during image assessment of the calcaneus method. Difficulties were grouped as those due to technical difficulties e.g. landmark(s) lying outside of the edge of the image; resolution issues, insufficient resolution to assess image easily; positioning—landmark not visible, or not clearly visible due to limb rotation or shadowing; and if rater was ‘unsure’ due to problems with method description.

**Fig 9:**
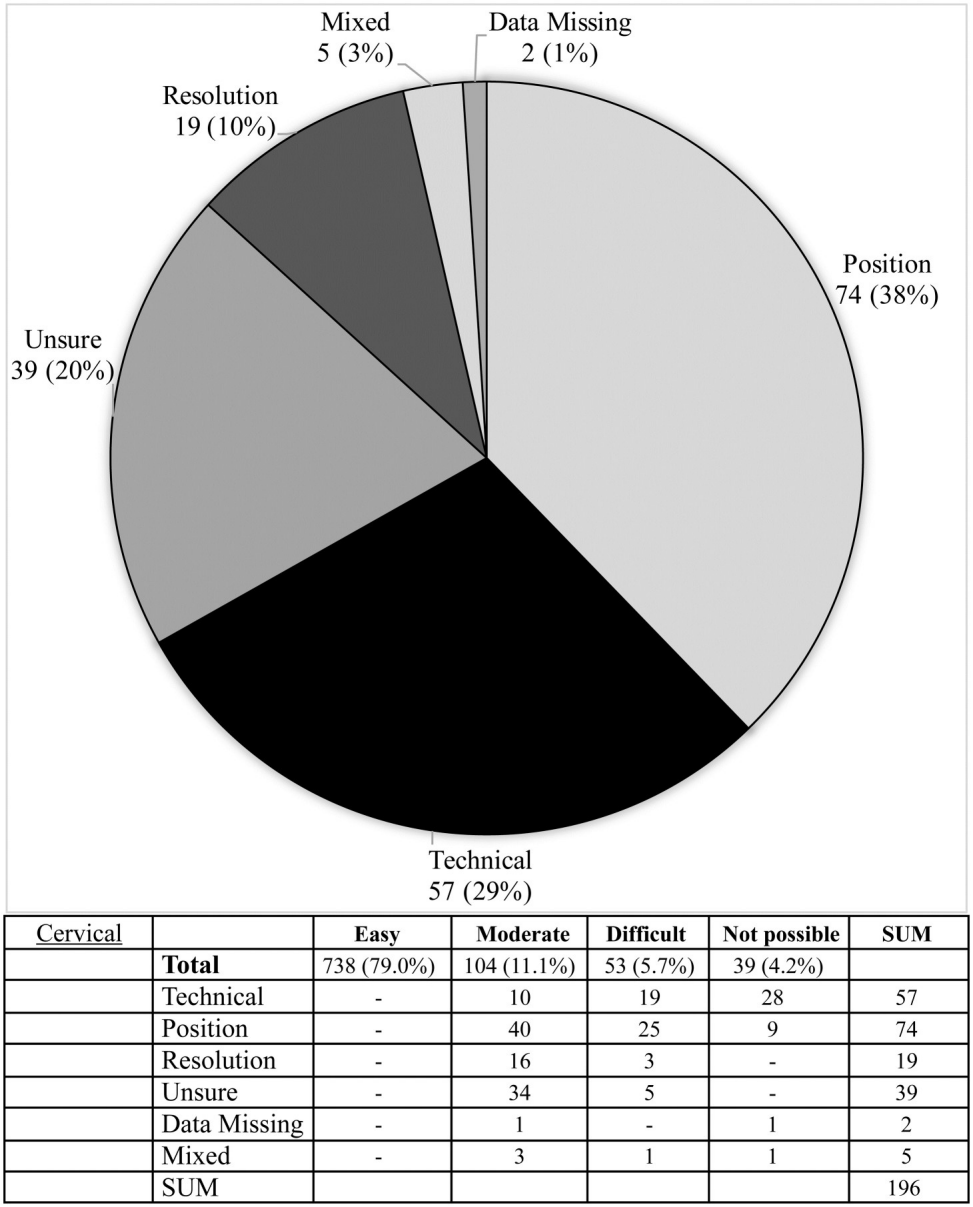
Difficulties reported during image assessment of the cervical method. Difficulties were grouped as those due to technical difficulties e.g. landmark(s) lying outside of the edge of the image; resolution issues, insufficient resolution to assess image easily; positioning—landmark not visible, or not clearly visible due to limb rotation or shadowing; and if rater was ‘unsure’ due to problems with method description.

**Fig 10:**
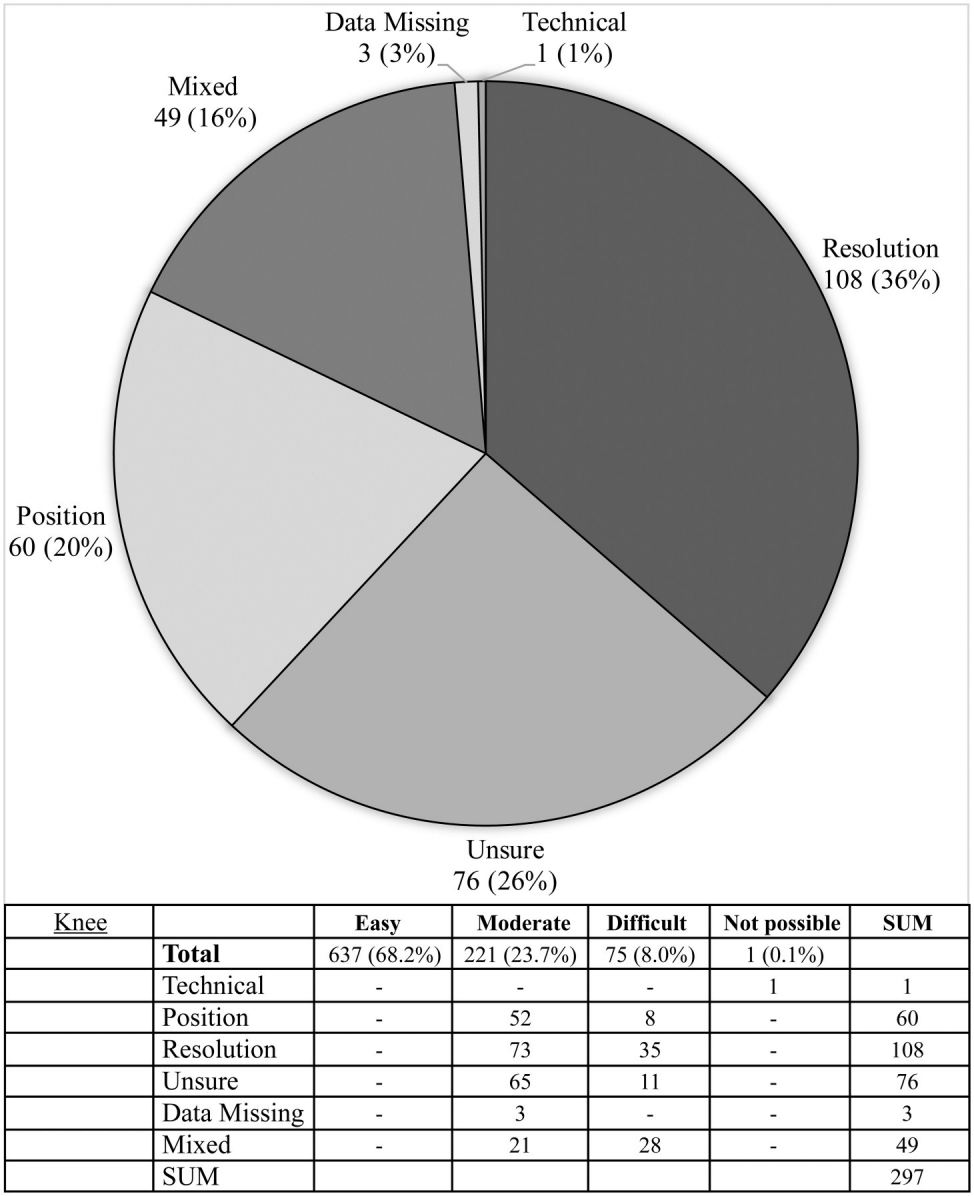
Difficulties reported during image assessment of the knee method. Difficulties were grouped as those due to technical difficulties e.g. landmark(s) lying outside of the edge of the image; resolution issues, insufficient resolution to assess image easily; positioning—landmark not visible, or not clearly visible due to limb rotation or shadowing; and if rater was ‘unsure’ due to problems with method description.

**Fig 11:**
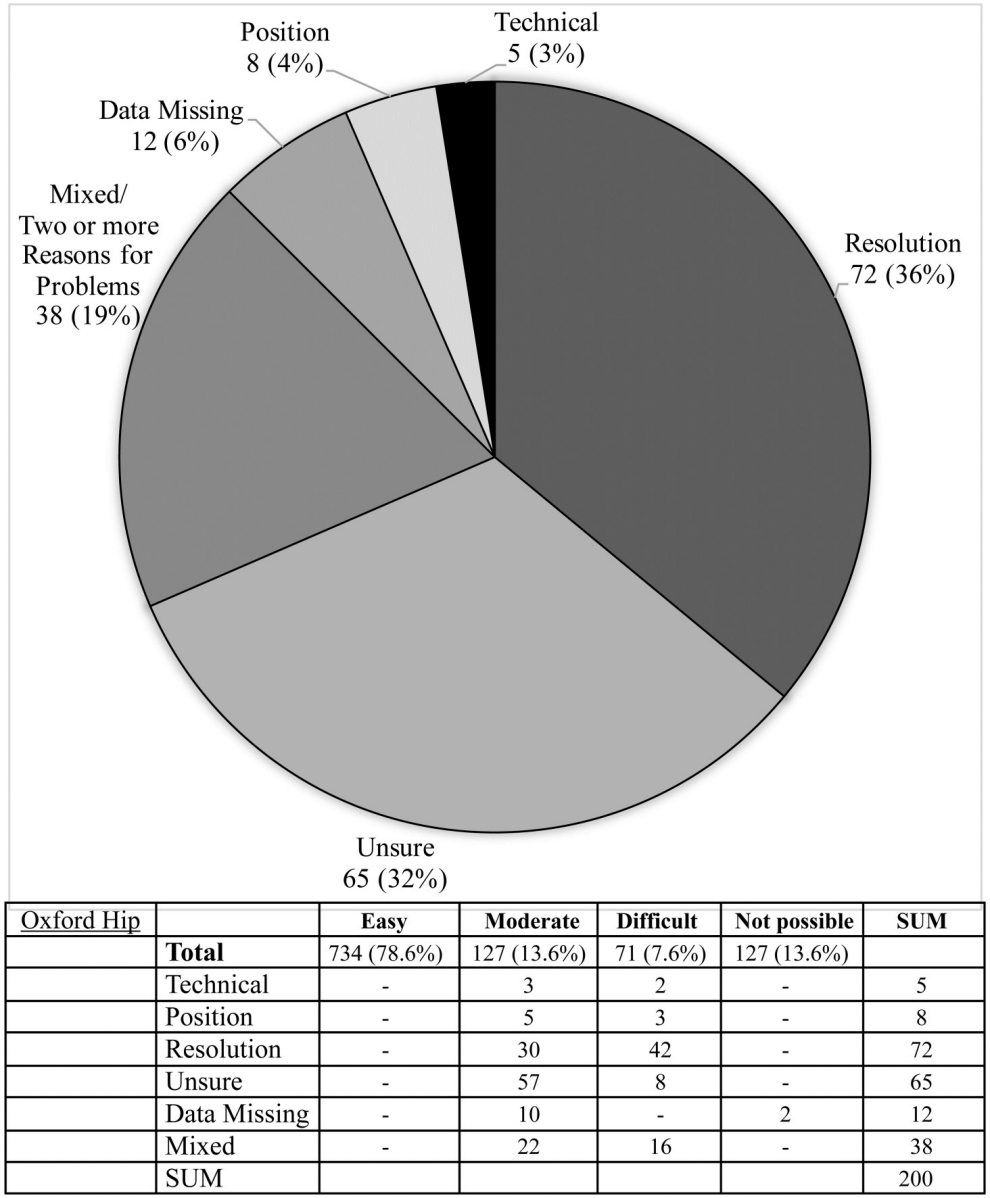
Difficulties reported during image assessment of the Oxford hip method. Difficulties were grouped as those due to technical difficulties e.g. landmark(s) lying outside of the edge of the image; resolution issues, insufficient resolution to assess image easily; positioning—landmark not visible, or not clearly visible due to limb rotation or shadowing; and if rater was ‘unsure’ due to problems with method description.

**Fig 12:**
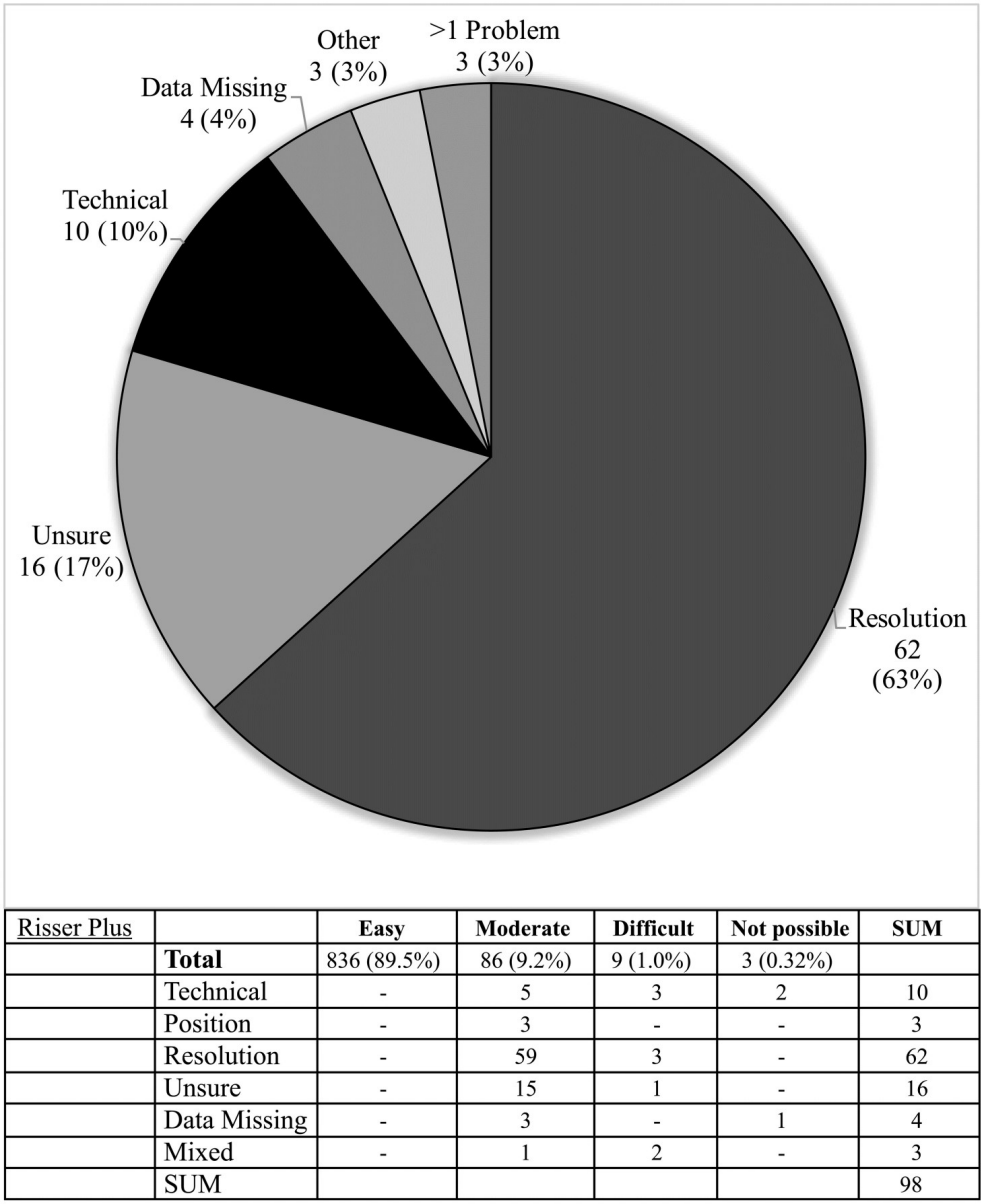
Difficulties reported during image assessment of the Risser plus method. Difficulties were grouped as those due to technical difficulties e.g. landmark(s) lying outside of the edge of the image; resolution issues, insufficient resolution to assess image easily; positioning—landmark not visible, or not clearly visible due to limb rotation or shadowing; and if rater was ‘unsure’ due to problems with method description.

The Risser ‘plus’ system received the most favourable ratings with 89.5% of scans (836/934) receiving an ‘easy’ rating and a further 9.2% of scans (82/934) rated ‘moderate’. Similarly, the Oxford hip method and cervical methods saw good ratings with 78.6% & 79.0% rated ‘easy’ and 13.6% and 11.1% rated ‘moderate’, respectively. The calcaneus method exhibited the highest number of unevaluable scans (6.2% or 58 scans) with the most common cause being that the feet were not imaged (49 scans) or that feet overlapped to an extent that made them unevaluable (seven scans) (see Figs 8–12, Table 2).

**Table 2:** Difficulty of evaluation with each bone age method.

	Easy	Moderate	Difficult	Impossible	Easy	Moderate	Difficult	Impossible
**Calcaneus**	661	132	83	58	70.8%	14.1%	8.9%	6.2%
**Cervical**	738	104	53	39	79.0%	11.1%	5.7%	4.2%
**Knee**	637	221	75	1	68.2%	23.7%	8.0%	0.10%
**Oxford Hip**	734	127	71	2	78.6%	13.6%	7.6%	0.20%
**Risser Plus**	836	86	9	3	89.5%	9.2%	1.0%	0.32%

Summary of observer scan ratings using 4-point Likert scale as follows: ‘Easy’; ‘Moderate’: some minor exposure problems or minor obstruction, but evaluation could confidently be made; ‘Difficult’: significant obstruction, image was partially cut off eg 1/3 or less of a landmark obscured or cut, such that assumption must be made; ‘Impossible’–landmark of interest outside of image or totally obstructed.

(The knee and oxford hip methods are composed of multiple landmarks and so when the overall scan was good but individual problems with landmarks were found. they were summed such that problems with 2 landmarks = rating of ‘2’ and ≥3 = rating of ‘3’. Similarly if difficulties were only reported with 1 of the 3 landmarks. the scan was rated as ‘2’).

The knee method received the lowest number of ‘easy’ ratings at 68.2% of scans (637/934) and 23.7% of scans (221/934) were reported to have moderate difficulty. Problems with the knee method were distributed almost equally between regions: local problems were reported in 39.6% of cases to be due to the femur, 36.5% tibia, and 23.9% fibula. Reported causes behind problematic scans (297) were predominantly: resolution (36.4%, 108/297), uncertainty (25.6%, 76/297), position (20.2%, 60/297) or other (16.8%, 50/297). Uncertainty was reported in 76 images (25.6% of problematic cases) as the original description was felt to be lacking in precise differentiation between the stages, particularly between Stages 3 and 4.

#### 3. Evaluation time

**Fig 13:**
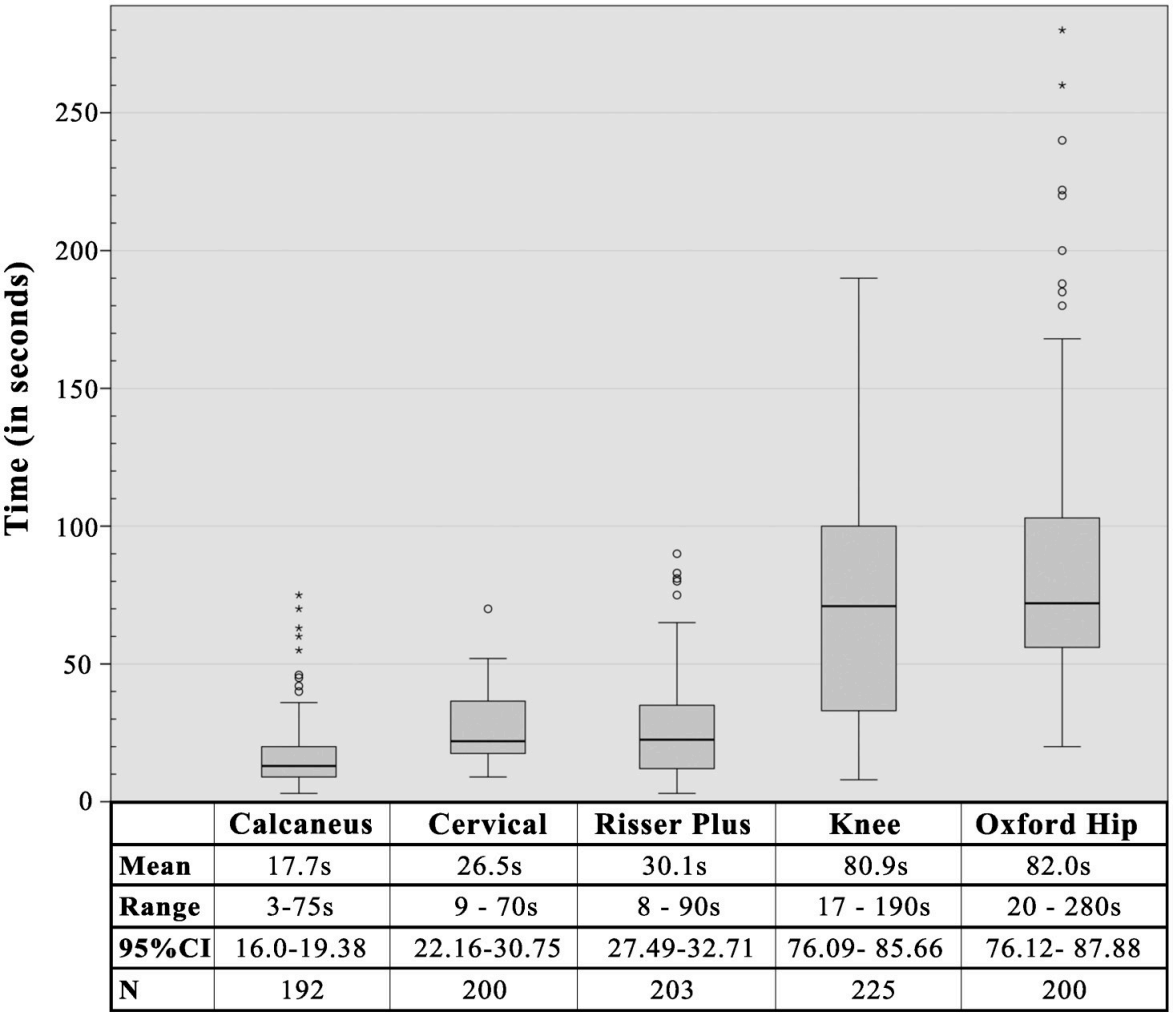
Timing data for each of the bone age methods.

Methods with fewer stages had shorter evaluation times. The six-stage calcaneus method was found to be the fastest at just 17.7 seconds, significantly quicker than the next fastest, the similarly six-stage cervical method’s 26.5s (independent t-test, p<0.05). The Oxford hip method was the slowest, sometimes taking more than four minutes due to the nine regions of interest to be evaluated, and uncertainty due to problems with lesser trochanter visibility (see discussion) (See Fig 13).

## Discussion

Since its’ introduction in 2007, the EOS Scanner has seen increasing popularity in clinics across Europe and North America with more than 300 systems installed in 34 countries [18]. Our study aimed to highlight alternatives to traditionally recommended systems for bone age estimation and to evaluate compatibility with the recommended patient position of the EOS scanner (Table 3). The use of alternative methods could allow us to avoid further radiation, as a hand and wrist X-ray exposes the child to a radiation of burden of 0.07 to 0.17 μSv depending on his/her age [19].

**Table 3:** Advantages and disadvantages of methods assessed.

	Calcaneus	Cervical	Knee	Oxford Hip	Risser Plus
**Reliability**	**Excellent (0.945)**	**Excellent (0.976)**	Good (0.865)	**Excellent (0.902)**	**Excellent (0.940)**
**Readability**	70.8% of scans easily assessable, 6.2% of scans unassessable **Image length affected readability**: Image must cover entire length of lower limb or not possible **Resolution**: can be difficulty distinguishing the timing of end of fusion (stage 4 vs. 5).	70.8% of scans easily assessable **Image length affected readability**: EOS image must cover entire length of cervical spine or not possible **Positioning:** (i) Head tilt can lead to mild difficulties. (ii)Hand position can obscure vertebrae making evaluation difficult or position (strict EOS protocol must be applied!)	68.2% of scans easily assessable **Step length:** can influence readability of lateral radiographs. **Resolution**: harder to evaluate features important in more mature stages (trabecular continuity, end of fusion)	78.6% of scans easily assessable **Complicated:** large no. of regions must be evaluated. **Modified Oxford:** simplified 5-region method may be superior for the clinician, however, omission of lesser trochanter may be required.	**89.5% of scans easily assessable (highest rated)**
**Speed**	**Fastest method** (17.1s)	Fast (26.5s)	Slower (80.9s)	Slowest method (82s)	Fast (30.1s)
**Age Range**	Broad age range: (4.32–11.03y)	Broad age range (4.73–13.57y)	Broad age range (5.07–15.02y)	**Broadest age range** (4.0–15.08 y)	Stages start later than other methods (6.55–15.27y)
**Other**	**Simple & Easy to learn** High rater satisfaction	High rater satisfaction	Low rater satisfaction	High rater satisfaction though time consuming.	High rater satisfaction **Familiar to orthopaedic physicians**

Reliability values listed above are intraclass coefficient estimates of inter-observer reliability, see Table 1 for intra-observer coefficient values. Age range describes the chronological of first recorded incidence of 2^nd^ stage within each system and the first incidence of the highest stage.

After assessment, three of the methods were more satisfactory and will be highlighted.

The Risser ‘plus’ system combines European and American Risser systems with tri-radiate cartilage evaluation and has been included in the recommendations of the Scoliosis Research Society since 2014 [14]. Our raters reported the highest number of ‘easy’ scans with this method (89.5%) and excellent reliability ICC scores. Furthermore, it was a relatively fast method (mean evaluation 30.1s). The Risser system without inclusion of tri-radiate cartilage evaluation is not recommended: Stage 0+ started at 6.55 years old with median 11.75, in comparison with 7.29 years old (or 11.53 years old when 1 outlier was excluded) at first presentation of Stage 1. Resolution was a moderate issue in 59 cases, predominantly due to difficulty with identifying the ending of fusion of the iliac apophysis—40 of these images were Risser 4 or 5. The number of unevaluable images was lower than that reported with Bone Xpert software, Martin et al. reported seven of 1097 images (0.64%) could not be initially evaluated due to insufficient image quality, contrast or size [20]. The Risser system had a rejection rate of 0.32% (3/938) and similarly low were the knee 0.11% (1/938), and Oxford hip methods 0.21% (2/938). The calcaneus and cervical methods had considerably higher numbers of unassessable scans (8.9%, 4.2%).

The calcaneus method, a method introduced in 2015 [7], returned mixed results. While difficulties were noted in a large number of scans (29.2% or 273/934), 52% of these were due to calcanei being partially or totally cut off at the time of image capture rather than due to difficulty with the scan itself. Foot positioning also caused difficulty, with overlap of the feet being the cause of 22.7% of those with moderate difficulty. That being said, this method was found to be the fastest (17.7s) and raters reported high levels of satisfaction, as it was easy to learn, use and remember. In the original description inclusion of plantar sesamoid evaluation is recommended, but due to the absence of the dorso-plantar plane and significant overlap between feet on the lateral image it was not felt to be reliable and it was not included in our assessment. The calcaneus method is based on the historical Greulich-Pyle ‘Brush’ population and Li et al recommended mild corrections for interpretation with modern age children, as they found Stage 3 and 4 girls were delayed by 0.64 and 0.58 years, respectively, compared to the historical population [21]. Our study showed similar delays, of 0.94 and 2.2 years in Stage 3 and 4 females, and even Stage 4 boys of 1.61 years, however as both these studies were retrospective, this is possibly in part due to an artificial elevation of values compared to serial studies in which the earliest scan at each bone age stage can be identified.

The Oxford hip method, first described in 1957 [13] consists of evaluation of nine different landmarks and as a result was the slowest method used with a mean 82s per evaluation. The 45-point scoring system however makes it a favorable instrument in a scientific setting, in which precision and a finer gradation of maturity is of more importance than time taken for evaluations. However, with experience the mean time taken reported by our raters was faster than the two most popular hand-wrist methods: the Tanner & Whitehouse 2 (TW2) method has been reported to take an average 7.8 minutes for evaluation, while the Greulich-Pyle method, is estimated to take 1.4 minutes per image [22, 23]. In 40.9% of scans (382/946), observers reported some degree of difficulty in evaluating the lesser trochanter, in comparison with the femoral head (8.7%) greater trochanter (8.4%), ilium (7.4%) and tri-radiate cartilage (2.8%). A modified version consisting of 5 landmarks has been described in risk assessment of slipped capital femoral epiphysis [24, 25] occurring in a contralateral limb. When we evaluated the method based on just these 5 parameters, a greater number of scans had favorable ratings (83.9% vs. 78.6% were rated ‘easy’, and 11.5% vs. 13.6% rated ‘moderate’). The inclusion of the lesser trochanter in this abbreviated method continued to cause problems, however, and so it’s omission may be considered, as other authors have suggested [24].

### Common problems encountered

#### Step length

While lower step length was reported subjectively to cause problems in assessment of the knee and calcaneus, only a mild inverse correlation was found with ratings when assessed by Spearman correlation (-0.100, p<0.05) in the case of the knee, and no significant correlation with the calcaneus (p = 0.202). A significantly lower average step length however was found in those where the lateral knee image was reported as unevaluable, due to the overlapping contralateral knee (58.31cm ± 46.95 vs. 78.45cm ±53.5)

#### Resolution

While the EOS image resolution is satisfactory for most structural evaluations, some of the features evaluated are very fine, and problems were specifically reported with: assessing trabecular continuity in the knee, and determining whether fusion was almost complete or had fully completed in calcaneus (Stage 4 vs. 5), knee (3 vs. 4) and femoral head (stage 6 vs. 7).

#### Image size

As a result of physician personal preference many of our images were not full body-length images, rather they excluded part or all of the upper cervical vertebrae (partly in 20, completely in 26 cases) or calcaneus (partly in 95 cases, completely in 49). Furthermore, the posterior calcaneal pole of the posterior foot often lay partially outside of the image. While this problem cannot be corrected retrospectively, ensuring that future scans include these areas is easily achievable.

#### Position

The cervical method was affected by variations in head and hand position in 77 scans. The most common causes were: lateral head tilt (37 images) leading to moderate (24), difficult (11) or impossible evaluations (2); hand/fingers partially covering the upper cervical vertebrae in 30 images (patients with an open hand often placed their thumb posteriorly, leading to shadowing over C2 and even C3 vertebrae) leading to moderate (13), difficult (11) or impossible evaluations (six).

This study has a number of limitations. Ratings are subjective judgements carried out by human observers in an effort to elucidate which methods are ‘better’ or ‘more suitable’–a hard concept to define. The rating system used may also have favoured methods that use more landmarks, as an ‘impossible’ rating was less likely in such cases. We endeavoured however to include our experiences and likely pitfalls when using each method to be more informative to the reader.

Jackson et al. recently reported on altering hand position to assess bone age in EOS images, however, they noted that this “may alter the spinal alignment and affect sagittal balance or shoulder height”, which was neither controlled form, nor measured [26]. In our clinic, attempts to alter upper limb position resulted in altered thoracic and cervical spine position and so were halted (unpublished data).

## Conclusions

Our findings supported the continued use of the Risser system but with the inclusion of tri-radiate assessment as per the recommendations of the Scoliosis Research Society. The Oxford hip method took the greatest time to apply, its fine scale and broad age range coverage suggests its use is appropriate for a research environment, although it may be simplified by omission of the lesser trochanter, as suggested by other authors [24]. While the calcaneus method was not always applicable for retrospective examination of our EOS images, it may serve to be a very useful and easy-to-remember alternative for maturity assessment, if efforts are made to ensure to capture the foot and calcaneus during image capture.

## Supporting information

S1 Fig Alternative bone age methods evaluated. Summaries of the six methods applied in pilot study. See original articles for full details of the individual methods. (All illustrations, unless otherwise stated, are reprinted from O’Sullivan et al. [17], creative commons license: https://creativecommons.org/licenses/by/4.0/legalcode). **(a) Cervical Bone Assessment as per Hassel & Farman (1995).** (Adapted with permission from Schlégl et al. [16]). *1*. *Initiation*: the inferior borders of C2, C3 and C4 are all flat. Upper borders taper from posterior to anterior giving the body a wedge shape. *2*. *Acceleration*: C2 and C3 develop concavities in their inferior borders, while that of C4 remains flat. Bodies of C3 and C4 are almost rectangular in shape. 3. *Transition*: Concavities in C2 and C3 are now deeper and distinct with C4 beginning to develop a concave inferior border too. Bodies of C3 and C4 are rectangular in shape. 4. *Deceleration*: C2, C3 and C4 all have distinct concavities in their inferior borders, and the bodies of C3 and C4 are becoming more square in shape. 5. *Maturation*: Concavities of C2, C3 and C4 are more accentuated in the inferior borders, and C3, C4 bodies are almost square or square in shape. 6. *Completion*: Deep concavities are found in the inferior borders of C2, C3 and C4 and the bodies are square or column-like, with a vertical dimension greater than their horizontal dimension. **(b) Shoulder Assessment as per Schaefer et al. (2015).** Assessment is performed on three regions of the shoulder and scores or ‘phases’ can be compared to age values from 10–24 years old. (i) Proximal Humerus—*1*. *Open union*: A continuous radiolucent line at the proximal humerus epiphyseal plate is visible. *2*. *Fusing*: Epiphyseal fusion is imminent, indicated by central haziness, or is in process. Peripheral radiolucent lines are visible; *3*. *Unfused notch*: Near-complete fusion with only peripheral notches visible, most commonly under the greater tubercle.; *4*. *Complete union*: No radiolucency remains. A radiopaque line may persist indicting the site of fusion. (ii) Acromion—*Phase 0*. *Not present*: No apophysis is observed. The acromion is marked by a rounded; *Phase 1 Present; open or fusing*: The apophysis is present and a radiolucent line is clearly visible. *Phase 4 Complete union*: No line of radiolucency is evident. (iii) Angle/apex: *Phase 1*. *Present; Phase 2*. *Open or fusing*: The apophysis is clearly visible sitting on top of, or at the tip of, the coracoid process. A radiolucent line is clearly visible. (Note: Only the proximal Humerus can be described by 4 distinct phases). **(c) Risser ‘Plus’ Method as per Negrini et al (2015).** *Risser 0-*: Open triradiate cartilage, *Risser 0+*: Fused triradiate cartilage, *Risser 1*: 10–25% coverage of iliac crest, *Risser 2*: 25–50% coverage, *Risser 3*: 50–75% coverage, *Risser 3/4*: 75–100% coverage, *Risser 4*: Fusion started, *Risser 5*: Fusion completed. **(d) Oxford Hip Method from Acheson (1957).** Nine regions are assessed as per pictorial below and values scores summed: femoral head, greater trochanter, lesser trochanter, iliac crest, ischium, ischio-pubic junction, pubic bone, acetabulum and tri-radiate cartilage. The five regions assessed in the abbreviated or ‘modified’ version of this method are marked with an asterisk (*) (Stasikelis et al. 1996 [24]). **(e) Knee from O’Connor et al. (2008).** *Stage 0*. *Non-Union*: clear radiolucent strip between both sides of epiphyseal plate, *Stage* 1. *Beginning Union*: very narrow radiolucent strip, central hazy/ blurring as fusion starts (<50% of gap); *Stage 2*. *Active Union*: ‘Capping’ as epiphysis overlap the metaphysis. Fusion line/ area of greater density area where fusion is taking place. Some areas of radiolucency remain, but fusion area is >50% of gap. *Stage 3*. *Recent Union*: Fine line of fusion remains but epiphysis and metaphysis are united (so-called ‘complete capping’). May be small notches at margins of <2mm. Discontinuity of trabeculae between former epiphysis and diaphysis. *Stage 4*. *Complete Union*: mature bone, with no notches at margins, and continuous trabeculae. A thin terminal line or ‘epiphyseal scar’ may remain at the site of the epiphyseal plate. **(f) Calcaneus. Adapted from Nicholson et al. 2015.** Stage 0: No ossification can be seen. Stage 1: The apophysis covers < 50% of the metaphysis. Stage 2: The apophysis covers ≥ 50% of the metaphysis but has not reached the plantar edge. Stage 3: Apophysis is within 2 mm of the plantar edge of the calcaneal concavity, as shown by the black arrow. Stage 4: Fusion taking place, but not yet complete as areas of radiolucency visible on dorsal and plantar edges. Fusion starts in the central area of the apophysis. Stage 5: Fusion is complete.
(TIF)

S1 Table Bone age methods in the scientific literature 1931–2016. Summary table of all bone age and dental age methods encountered during literature review. The website pubmed.gov was accessed on 2016.03.30 and searched using keywords "bone age", "skeletal age" and "skeletal maturation" without any restrictions on date or language. After 433 duplicates were removed, all 4758 abstracts were reviewed for bone age methods used and articles accessed if not listed in the abstract. Original articles describing each method were sought if not included in the original list. Citation number as per google scholar (scholar.google.com) were collected at the time of preparing this table (2019.10.09). ’*Article not found’*: In some cases the article could not be found despite attempts to locate it, *’Foreign Language [language]’*: search did not exclude foreign language inclusions, as a result some lesser known methods are included, which were not described in English, and could not be located. Three methods included were described after the original search date(marked *), however due to their potential future interest to bone age investigators they have been included. One method was included that was not returned in the search (marked †), but encountered during the course of the research and was included in the interest of completeness. (AP: Anteroposterior, CT: Computed tomography, GP: Greulich-Pyle Atlas, HF: Hassel-Farman method, mo: months, MR: Magnetic resonance, y: years).
(DOCX)
