# Supplementary figures and images for: Alternative methods for skeletal maturity estimation with the EOS scanner—Experience from 934 patients
Source: PLoS One. 2022 May 6;17(5):e0267668. doi: 10.1371/journal.pone.0267668 (PMC9075679; doi:10.1371/journal.pone.0267668)

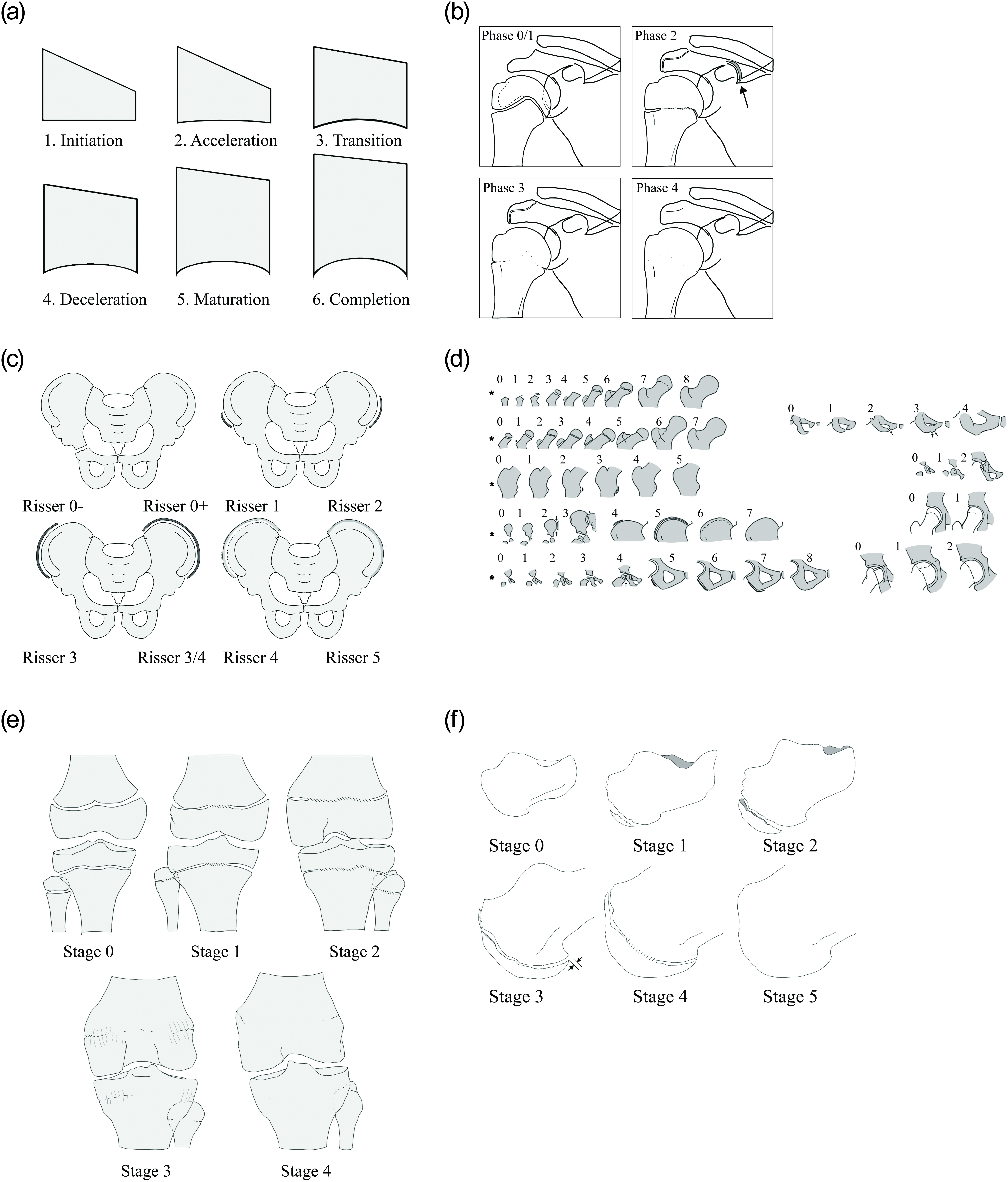

Supplement: S1 Fig — Summaries of the six methods applied in pilot study. See original articles for full details of the individual methods. (All illustrations, unless otherwise stated, are reprinted from O’Sullivan et al. [17], creative commons license: https://creativecommons.org/licenses/by/4.0/legalcode). (a) Cervical Bone Assessment as per Hassel & Farman (1995). (Adapted with permission from Schlégl et al. [16]). 1. Initiation: the inferior borders of C2, C3 and C4 are all flat. Upper borders taper from posterior to anterior giving the body a wedge shape. 2. Acceleration: C2 and C3 develop concavities in their inferior borders, while that of C4 remains flat. Bodies of C3 and C4 are almost rectangular in shape. 3. Transition: Concavities in C2 and C3 are now deeper and distinct with C4 beginning to develop a concave inferior border too. Bodies of C3 and C4 are rectangular in shape. 4. Deceleration: C2, C3 and C4 all have distinct concavities in their inferior borders, and the bodies of C3 and C4 are becoming more square in shape. 5. Maturation: Concavities of C2, C3 and C4 are more accentuated in the inferior borders, and C3, C4 bodies are almost square or square in shape. 6. Completion: Deep concavities are found in the inferior borders of C2, C3 and C4 and the bodies are square or column-like, with a vertical dimension greater than their horizontal dimension. (b) Shoulder Assessment as per Schaefer et al. (2015). Assessment is performed on three regions of the shoulder and scores or ‘phases’ can be compared to age values from 10–24 years old. (i) Proximal Humerus—1. Open union: A continuous radiolucent line at the proximal humerus epiphyseal plate is visible. 2. Fusing: Epiphyseal fusion is imminent, indicated by central haziness, or is in process. Peripheral radiolucent lines are visible; 3. Unfused notch: Near-complete fusion with only peripheral notches visible, most commonly under the greater tubercle.; 4. Complete union: No radiolucency remains. A radiopaque line may persist [file pone.0267668.s001.tif]
