# Supplementary material for: Alternative methods for skeletal maturity estimation with the EOS scanner—Experience from 934 patients
Source: PLoS One. 2022 May 6;17(5):e0267668. doi: 10.1371/journal.pone.0267668 (PMC9075679; doi:10.1371/journal.pone.0267668)
Supplement: S1 Table — Summary table of all bone age and dental age methods encountered during literature review. The website pubmed.gov was accessed on 2016.03.30 and searched using keywords "bone age", "skeletal age" and "skeletal maturation" without any restrictions on date or language. After 433 duplicates were removed, all 4758 abstracts were reviewed for bone age methods used and articles accessed if not listed in the abstract. Original articles describing each method were sought if not included in the original list. Citation number as per google scholar (scholar.google.com) were collected at the time of preparing this table (2019.10.09). ’Article not found’: In some cases the article could not be found despite attempts to locate it, ’Foreign Language [language]’: search did not exclude foreign language inclusions, as a result some lesser known methods are included, which were not described in English, and could not be located. Three methods included were described after the original search date(marked *), however due to their potential future interest to bone age investigators they have been included. One method was included that was not returned in the search (marked †), but encountered during the course of the research and was included in the interest of completeness. (AP: Anteroposterior, CT: Computed tomography, GP: Greulich-Pyle Atlas, HF: Hassel-Farman method, mo: months, MR: Magnetic resonance, y: years). (DOCX) [file pone.0267668.s002.docx]

**S1 Table. Bone age methods in the scientific literature 1931 - 2016.** Summary table of all bone age and dental age methods encountered during literature review. The website pubmed.gov was accessed on 2016.03.30 and searched using keywords "bone age", "skeletal age" and "skeletal maturation" without any restrictions on date or language. After 433 duplicates were removed, all 4758 abstracts were reviewed for bone age methods used and articles accessed if not listed in the abstract. Original articles describing each method were sought if not included in the original list. Citation number as per google scholar (scholar.google.com) were collected at the time of preparing this table (2019.10.09). '*Article not found':* In some cases the article could not be found despite attempts to locate it, *'Foreign Language [language]':* search did not exclude foreign language inclusions, as a result some lesser known methods are included, which were not described in English, and could not be located. Three methods included were described after the original search date(marked *), however due to their potential future interest to bone age investigators they have been included. One method was included that was not returned in the search (marked †), but encountered during the course of the research and was included in the interest of completeness. (AP: Anteroposterior, CT: Computed tomography, GP: Greulich-Pyle Atlas, HF: Hassel-Farman method, mo: months, MR: Magnetic resonance, y: years)

| Region | Author | Year of Pub. | Type | Citations | Modality | Image type | No. of Stages | Summary of Method | Reference |
| --- | --- | --- | --- | --- | --- | --- | --- | --- | --- |
| Ankle & Foot | Hoerr, Pyle, Francis | 1962 | Atlas | 5 | X-ray | Lateral & AP  Foot | 31 ages | Atlas method of ankle and foot. | Hoerr NL, Pyle SI, Francis CC. Radiological Atlas of the Foot and Ankle. Charles C Thomas, Springfield,. 1962. |
| Calcaneus | Nicholson | 2015 | Scoring | 20 | X-ray | Lateral foot | 6 stages | Calcaneal apophysis is scored based on presence, extent and state of fusion. | Nicholson AD, Liu RW, Sanders JO, Cooperman DR. Relationship of Calcaneal and Iliac Apophyseal Ossification to Peak Height Velocity Timing in Children. J Bone Jt Surg. 2015;97(2):147-154. |
|  | Sherif | 2003 | Scoring | 3 | Ultrasound | Ultrasound | Linear scale | Normal range of calcaneal volume values in a 0-6 year old Egyptian population have been presented, as measured by ultrasound. | Sherif H, Noureldin M, Bakr AF, Mahfouz AE. Sonographic Measurement of Calcaneal Volume for Determination of Skeletal Age in Children. J Clin Ultrasound. 2003;31(9):457-460. |
| Cervical | Alhadlag & Al-shayea | 2013 | Scoring | 6 | X-ray | Lateral cephalogram | 5 stages | Angular based method: user traces C2-C3-C4 and calculates the angles between a line running along the base, and the highest point of the inferior concavity. 5 stages were identified correlating to Baccetti's method. Only males. | Alhadlaq AM, Al-shayea EI. New method for evaluation of cervical vertebral maturation based on angular measurements. Saudi Med J. 2013;33(4):388-394. |
|  | Baccetti: 6-stage | 2002 | Scoring | 655 | X-ray | Lateral cephalogram | 6 stages | Second to fourth cervical vertebrae are assessed by morphology: height, inferior curvature and shape. “CS” or “cervical stage” method. | Baccetti T, Franchi L, McNamara Jr JA. An improved version of the cervical vertebral maturation (CVM) method for the assessment of mandibular growth. Angle Orthod. 2002;72(4):316-323. |
|  | Baccetti modified: Semi-automated | 2012 | Scoring | 11 | X-ray | Lateral cephalogram | 6 stages | User selects 13 landmarks on C2-C4 indicating anterior inferior, posterior inferior, anterior superior (C3 & C4 only) and posterior superior points (C3 & C4 only), and the highest point of the inferior concave. Stages as per Baccetti are then applied using computer software. | Baptista RS, Quaglio CL, Mourad LMEH, et al. A semi-automated method for bone age assessment using cervical vertebral maturation. Angle Orthod. 2012;82(4):658-662. |
|  | Baccetti modified: CT | 2015 | Scoring | 29 | CT | Cone-beam CT | 6 stages | Lateral cephalogram generate from CBCT and evaluated. | Angelieri F, Franchi L, Cevidanes LHS, McNamara Jr JA. Diagnostic performance of skeletal maturity for the assessment of midpalatal suture maturation. Am J Orthod Dentofac Orthop. 2015;148(6):1010-1016. |
|  | Baccetti modified: 5 stage | 2006 | Scoring | 23 | X-ray | Lateral cephalogram | 5 stages | Sixth stage is not included. | Santos ECA, Bertoz FA, Arantes FDM, Reis PMP, Bertoz APDM. Skeletal maturation analysis by morphological evaluation of the cervical vertebrae. J Clin Pediatr Dent. 2006;30(3):265-270. |
|  | Caldas | 2007 | Scoring | 42 | X-ray | Lateral cephalogram | Linear regression equation | Based on a formula, reader traces and then measures height, width, angle etc. | Caldas M de P, Ambrosano GMB, Haiter Neto F. New formula to objectively evaluate skeletal maturation using lateral cephalometric radiographs. Braz Oral Res. 2007;21(4):330-335. |
|  | Caldas modified: Semi-automated | 2010 | Scoring | 25 | X-ray | Lateral cephalogram | Linear regression equation | Based on a formula, selects landmarks indicating anterior/posterior and superior/inferior poles, and software calculates bone age based on Caldas' equation. | Caldas M de P, Bovi Ambrosano GM, Neto FH. Computer-assisted analysis of cervical vertebral bone age using cephalometric radiographs in Brazilian subjects. Braz Oral Res. 2010;24(1):120-126. |
|  | Chen | 2008 | Scoring | 64 | X-ray | Lateral cephalogram | 4 stages | So-called "Quantitative cervical morphology" or QVCM. Tracings of C3, C4: ant height, posterior height, middle height and superior/midline/inferior anteroposterior length. Formula made based on these can calculate SMI (as used in Fishman maturity) | Chen L-L, Xu T-M, Jiang J-H, Zhang X-Z, Lin J-X. Quantitative cervical vertebral maturation assessment in adolescents with normal occlusion: a mixed longitudinal study. Am J Orthod Dentofac Orthop. 2008;134(6):720-e1. |
|  | Franchi | 2000 | Atlas | 420 | X-ray | Lateral cephalogram | 6 stages | Cervical vertebral morphology are assessed at C2-C6. Baccetti et al. method is based on a abbreviated system. | Franchi L, Baccetti T, McNamara JA. Mandibular growth as related to cervical vertebral maturation and body height. Am J Orthod Dentofac Orthop. 2000;118(3):335-340. |
|  | Harfin | 2008 | - | 3 | X-ray | Lateral cephalogram | - | Not found - foreign language [Polish] | Harfin, J.F., Kahn de Gruner, S.E., Porta, G. and Kaplan, A., 2008. Nowy sposób określania wieku szkieletowego oparty na wtórnych ośrodkach kostnienia kręgów szyjnych. In Forum Ortod (Vol. 4, No. 2, pp. 33-43). |
|  | Hassel-Farman | 1995 | Scoring | 713 | X-ray | Lateral cephalogram | 6 stages | C2-C4 morphology based on shape, inferior curve depth, and height. | Hassel B, Farman AG. Skeletal maturation evaluation using cervical vertebrae. Am J Orthod Dentofacial Orthop. 1995;107(1):58-66.. |
|  | Hassel-Farman modification: C3 only | 2005 | Scoring | 26 | X-ray | Lateral cephalogram | 6 stages | HF method, only assessing C3. | Seedat AK, Forsberg CD. An evaluation of the third cervical vertebra (C3) as a growth indicator in Black subjects. SADJ. 2005 May;60(4):156,158-60 |
|  | Hassel-Farman modification: Semi-automated | 2015 | Scoring | 3 | X-ray | Lateral cephalogram | 6 stages | Software assisted. Physician traces the vertebrae, and it helps estimate. | Dzemidzic, V., Sokic, E., Tiro, A. and Nakas, E., 2015. Computer based assessment of cervical vertebral maturation stages using digital lateral cephalograms. Acta Informatica Medica, 23(6), p.364. |
|  | Hassel-Farman modification: 3D-CT | 2016 | Scoring | 6 | CT | Lateral image  3D CT | 6 stages | HF method, using lateral aspect as generated from 3D CT images. | Bonfim MAE, Costa ALF, Fuziy A, Ximenez MEL, Cotrim-Ferreira FA, Ferreira-Santos RI. Cervical vertebrae maturation index estimates on cone beam CT: 3D reconstructions vs sagittal sections. Dentomaxillofacial Radiol. 2016;45(1):20150162. |
|  | Lamparski | 1972 | Scoring | 17 | X-ray | Lateral cephalogram | 6 stages | First cervical morphology method described. | ﻿Lamparski DG. Skeletal age assessment utilizing cervical vertebrae. 1975;67(4):458-459. |
|  | Mito | 2002 | Scoring | 180 | X-ray | Lateral cephalogram | Linear regression equation | C3 & C4 tracings made and measurements of anterior height, posterior height, midpoint height and anteroposterior length. Equation made based on these. | Mito T, Sato K, Mitani H. Cervical vertebral bone age in girls. Am J Orthod Dentofac Orthop. 2002;122(4):380-385. |
|  | Rhee | 2015 | Scoring | 2 | CT | Cone-beam CT | Linear regression equation | C2-C4: 8 points per vertebra, 6 points on odontoid process. Linear regression generated equations | Rhee CH, Shin SM, Choi YS, et al. Application of statistical shape analysis for the estimation of bone and forensic age using the shapes of the 2nd, 3rd, and 4th cervical vertebrae in a young Japanese population. Forensic Sci Int. 2015;257:513.e1-513.e9. |
|  | San Roman | 2002 | Scoring | 254 | X-ray | Lateral cephalogram | Linear regression equation | C3 & C4: equation based on stages of 3 features: inferior concavity (6 stages), vertebral shape (6 stages) and height vs. width (6 stages). | Román PS, Palma JC, Oteo MD, Nevado E. Skeletal maturation determined by cervical vertebrae development. Eur J Orthod. 2002;24(3):303-311. |
|  | Santiago | 2014 | Scoring | 5 | X-ray | Lateral cephalogram | 4 stages | User selects 13 landmarks on C2-C4 indicating anterior inferior, posterior inferior, anterior superior (C3 & C4 only) and posterior superior points (C3 & C4 only), and the highest point of the inferior concave. Stages as per Baccetti are then applied using computer software. Stages correlated to Fishman SMI 1:1-3, 2: 4-7, 3: 8-9, 4:10-11. | Santiago RC, Cunha AR, Júnior GC, et al. New software for cervical vertebral geometry assessment and its relationship to skeletal maturation-a pilot study. Dentomaxillofacial Radiol. 2014;43(2). |
|  | Su | 2006 | Scoring | 0 | - | - | Linear regression equation | Description in Chinese: Abstract - equation based on vertebral anterior height, height, anteroposterior length of C3 and C4. | Su L, Lü Y, Wang HM. Cervical vertebral bone age during puberty. Zhonghua kou qiang yi xue za zhi= Zhonghua kouqiang yixue zazhi= Chinese J Stomatol. 2006;41(12):728-729. |
|  | Varshosaz | 2012 | Scoring | 9 | X-ray | Lateral cephalogram | Linear regression equation | Anterior height of C4 measured and used as part of equation. 91 Iranian individuals aged 8-18 years | Varshosaz M, Ehsani S, Nouri M, Tavakoli MA. Bone age estimation by cervical vertebral dimensions in lateral cephalometry. Prog Orthod. 2012;13(2):126-131. |
|  | Yang | 2014 | Scoring | 8 | CT | Cone-beam CT | Linear regression equation | C1-C4: Axial images generated and 23 points selected across the vertebrae, (most anterior point, posterior point etc). Linear regression derived equations could then be applied to establish bone age. Equations not shown in paper, though they find several regions alone were correlated well with maturation. | Yang Y, Lee J, Kim Y, Cho B, Park S. Axial cervical vertebrae‐based multivariate regression model for the estimation of skeletal‐maturation status. Orthod Craniofac Res. 2014;17(3):187-196. |
| Clavicle | Kreitner | 1998 | Scoring | 249 | CT | Multi-slice CT | 3 stages | Appearance, partial fusion and complete fusion of medial epiphyseal ossification center. | Kreitner K-F, Schweden FJ, Riepert T, Nafe B, Thelen M. Bone age determination based on the study of the medial extremity of the clavicle. Eur Radiol. 1998;8(7):1116-1122. |
|  | Schmeling: 5 stage | 2004 | Scoring | 296 | X-ray | Chest X-ray | 5 stages | Initial 4 stages based on common schema: non-fused, fusing, almost fused, fused, in addition to a further fifth stage when no scar can be seen. | Schmeling A, Schulz R, Reisinger W, Mühler M, Wernecke K-D, Geserick G. Studies on the time frame for ossification of the medial clavicular epiphyseal cartilage in conventional radiography. Int J Legal Med. 2004;118(1):5-8. |
|  | Schmeling modified: 4 stage | 2015 | Scoring | 18 | CT | Multi-slice CT | 4 stages | Initial 4 stages based on common schema: non-fused, fusing, almost fused, fused (independent of presence or absence of scar) some studies such as Zhang et al. have used this 4 stage method. | Zhang K, Chen X, Zhao H, Dong X, Deng Z. Forensic Age Estimation Using Thin‐Slice Multidetector CT of the Clavicular Epiphyses Among Adolescent Western Chinese. J Forensic Sci. 2015;60(3):675-678. |
|  | Schmeling modified: MR | 2010 | Scoring | 87 | MR | MR | 5 stages | Initial 4 stages based on common schema: non-fused, fusing, almost fused, fused, in addition to a further stage 5 when no scar can be seen. | Hillewig E, De Tobel J, Cuche O, Vandemaele P, Piette M, Verstraete K. Magnetic resonance imaging of the medial extremity of the clavicle in forensic bone age determination: A new four-minute approach. Eur Radiol. 2011;21(4):757-767. |
|  | Schmeling modified: 4 stage | 2010 | Scoring | 179 | CT | Thin slice CT | 9 stages | Second and third stages are expanded with 3 substages each. | Kellinghaus M, Schulz R, Vieth V, Schmidt S, Pfeiffer H, Schmeling A. Enhanced possibilities to make statements on the ossification status of the medial clavicular epiphysis using an amplified staging scheme in evaluating thin-slice CT scans. Int J Legal Med. 2010;124(4):321-325. |
|  | Schmidt | 2007 | Scoring | 133 | MR | MR | 4 stages | Presence of ossification at the secondary center, extent and completion are assessed at the medial clavicle. | Schmidt S, Mühler M, Schmeling A, Reisinger W, Schulz R. Magnetic resonance imaging of the clavicular ossification. Int J Legal Med. 2007;121(4):321-324. |
|  | Schulz | 2008 | Scoring | 99 | Ultrasound | Ultrasound | 4 stages | Appearance and fusion of medial clavicle ossification centre are assessed, in addition to epiphyseal plate shape and state of ossification. | Schulz R, Zwiesigk P, Schiborr M, Schmidt S, Schmeling A. Ultrasound studies on the time course of clavicular ossification. Int J Legal Med. 2008;122(2):163-167. |
| Cranial | Lottering | 2015 | Scoring | 22 | CT | Multi-slice Head CT | 6 stages | 6 stages based on Spheno-Occipital Synchondrosis ossification. | Lottering N, MacGregor DM, Alston CL, Gregory LS. Ontogeny of the spheno‐occipital synchondrosis in a modern Queensland, Australian population using computed tomography. Am J Phys Anthropol. 2015;157(1):42-57. |
|  | Lottering | 2016 | Scoring | 9 | CT | Cranial/ cervical multi-slice CT | 24 stages, different regions (total 19). | 6 fontanelles & osteosynchondrosis regions are assessed. The time of closure of each region is described by as few as 2 or as many as 4 stages. | Lottering N, Macgregor DM, Alston CL, Watson D, Gregory LS. Introducing Computed Tomography Standards for Age Estimation of Modern Australian Subadults Using Postnatal Ossification Timings of Select Cranial and Cervical Sites. J Forensic Sci. 2016;61(3):39-52. |
|  | Bassed | 2010 | Scoring | 74 | CT | Multi-slice CT | 5 stages | Ossification extent from superior to inferior of the spheno-occipital chondrosis. (Staging modified from Powell and Brodie, 1963 - forensic study with X-ray). | Bassed RB, Briggs C, Drummer OH. Analysis of time of closure of the spheno-occipital synchondrosis using computed tomography. Forensic Sci Int. 2010;200(1-3):161-164. |
|  | Shirley & Jantz | 2011 | Scoring | 78 | CT | Cone-beam head CT | 3 stages | The open/fusing/fused state is assessed at the spheno-occipital osteochondrosis. | Shirley NR, Jantz RL. Spheno-occipital synchondrosis fusion in modern Americans. J Forensic Sci. 2011;56(3):580-585. |
|  | Franklin & Flavel | 2014 | Scoring | 34 | CT | Multi-detector CT | 4 stages | Spheno-occipital osteochondrosis is evaluated for open/fusing/fused with scar/ fused with no scar. | Franklin D, Flavel A. Brief Communication: Timing of spheno-occipital closure in modern Western Australians. Am J Phys Anthropol. 2014;153(1):132-138. |
|  | Ertürk | 1968 | Scoring | 5 | X-ray | Lateral cephalogram | No stages | Frontal sinus maximum dimension height and width measured are measured and can be compared against standards. | Ertürk, N., 1968. Teleroentgen studies on the development of the frontal sinus. Fortschritte der Kieferorthopadie, 29(2), pp.245-248. |
| Dental | Cameriere | 2006 | Scoring | 247 | X-ray | Panoramic X-ray | Linear regression equation | Open root apices of the teeth are measured, and summed. Data can be entered into linear regression equation. | Cameriere R, Ferrante L, Cingolani M. Age estimation in children by measurement of open apices in teeth. Int J Legal Med. 2006;120(1):49-52. |
|  | Demirjian | 1973 | Scoring | 2031 | X-ray | Panoramic X-ray | 63 stages | Seven left mandibular permanent teeth are rated in the order of the second molar, first molar, second premolar, first premolar, canine, lateral incisors and central incisor into eight stages [A-H] of tooth mineralization, stage 0 marks absence/ non-appearance of a tooth. All ratings are compared against a table to give a maturity score per tooth, which are summed, and then overall maturity score can be compared against a developmental percentile chart for each gender. | Demirjian A, Goldstein H, Tanner JM. Demirjian, A., A New System of Dental Age Assessment, Human Biology, 45:2 (1973:May) p.211. Hum Biol. 1973;45(2):211-227. |
|  | Demirjian modified: Chaillet | 2005 | Scoring | 158 | X-ray | Panoramic X-ray | 63 stages | "Multi-ethnic weighted score" chart, that allows use independent of knowledge of ethnicity. | Chaillet N, Nyström M, Demirjian A. Comparison of Dental Maturity in Children of Different Ethnic Origins: International Maturity Curves for Clinicians. J Forensic Sci. 2005;50(5):1-11. |
|  | Demirjian modified: Third Molar only | 1993 | Scoring | 500 | X-ray | Panoramic X-ray | 8 stages | Third molar only. All 4 evaluated and averaged. | Mincer HH, Harris EF, Berryman HE. The A.B.F.O. Study of Third Molar Development and Its Use as an Estimator of Chronological Age. J Forensic Sci. 1993;38(2):13418J. doi:10.1520/jfs13418j |
|  | Demirjian modified: Willems | 2001 | Scoring | 417 | X-ray | Panoramic X-ray | 63 stages | Updated chart for Belgian children to avoid overestimation of bone age. | Willems G, Van Olmen A, Spiessens B, Carels C. Dental age estimation in Belgian children: Demirjian’s technique revisited. J Forensic Sci. 2001;46(4):893-895. |
|  | Demirjian modified: Four Tooth Modification | 1989 | Scoring | 70 | X-ray | Panoramic X-ray | 36 stages | Using just 4 teeth. Based on Finnish standards. | Kataja M, Nyström M, Aine L. Dental maturity standards in southern Finland. Proc Finn Dent Soc. 1989;85(3):187-197. |
|  | Gat | 1972 | Scoring | 2 | X-ray | Panoramic X-ray | 84 stages | Fourteen permanent teeth on mandible and maxilla are scored from 1-5, excluding the third molar, and the sum of the values is the 'dental age'. | Gat H. An evaluation of dental ages of Norwegian children from the Bergen area. Univ Bergen, Bergen. 1972. |
|  | Gleiser & Hunt | 1995 | Scoring | 442 | X-ray | Lateral mandibular | 15 stages | First right mandibular permanent molar evaluated. | Gleiser I, Hunt Jr EE. The Permanent Mandibular First Molar: Its Calcification, Eruption and Decay. Am J Phys Anthropol. 1995;(13):253-283. |
|  | Gleiser & Hunt modification: Kullman | 1992 | Scoring | 251 | X-ray | Panoramic X-ray | 7 stages | Third molar root development is evaluated. | Kullman L, Johanson G, Akesson L. Root development of the lower third molar and its relation to chronological age. Swed Dent J. 1992;16(4):161-167. |
|  | Gleiser & Hunt modification: Liversidge | 2008 | Scoring | 157 | X-ray | Panoramic X-ray/ Supplementary apical in some cases | 15 stages | Third molar evaluated based on progressive stages described by changes in the crypt, cusp, crown, cleft, root and apex: applicable on from 16 years and above. | Liversidge HM. Timing of human mandibular third molar formation. Ann Hum Biol. 2008;35(3):294-321. |
|  | Gustafson & Koch | 1974 | Scoring | 432 | X-ray | Panoramic X-ray | 90 stages | Emergence and 3 maturation stages for right lower and left upper teeth are shown in their diagrams. Values are compared against a reference chart which gives weighting, and corresponding dental age. | Gustafson G, Koch G. Age estimation up to 16 years of age based on dental development. Odontol Revy. 1974;25(3):297-306. |
|  | Haavikko | 1974 | Scoring | 203 | X-ray | Panoramic X-ray | 6 stages | Upper and lower first molar, canine, 1st incisor and lower 2nd molar, 1st premolar and 2nd incisor are scored, and averaged. This can then allow division into 6 groups. | Haavikko K. Tooth formation age estimated on a few selected teeth. Proc Finn Dent Soc. 1974;70:15-19. |
|  | Leinonen | 1972 | Scoring | 10 | X-ray | Panoramic X-ray | 8 stages | Staging applied 0-7 of each mandibular tooth, the two sides were averaged. | Leinonen A, Wasz-Höckert B, Vuorinen P. Usefulness of the dental age obtained by orthopantomography as an indicator of the physical age. Proc Finn Dent Soc. 1972;68(5):235-242. |
|  | Liljeqvist & Lundberg | 1971 | Scoring | 172 | X-ray | Full oral series | 8 stages per tooth | 8 weighted stages applied based on dental morphology of crown, root etc, all teeth evaluated excepting upper pre-molar and molars. | Liliequist B, Lundberg M. Skeletal and tooth development: a methodologic investigation. Acta Radiol Diagnosis. 1971;11(2):97-112. |
|  | Moorrees | 1963 | Scoring | 2148 | X-ray | Lateral & intraoral radiographs (two population samples combined therefore differing radiograph types) | 14 stages | Mandibular teeth evaluated based on progressive stages described by changes in the crypt, cusp, crown, cleft, root and apex. | Moorrees CFA, Fanning EA, Hunt EE. Age Variation of Formation Stages for Ten Permanent Teeth. J Dent Res. 1963;42(6):1490-1502. |
|  | Moorrees modification: Anderson | 1976 | Scoring | 332 | X-ray | Panoramic Xray | 14 stages | Mandibular and maxillary teeth assessed, applied to as many teeth as possible and the score averaged. They provided alternative tables that some authors prefer. | Anderson DL, Thompson GW, Popovich F. Age of attainment of mineralization stages of the permanent dentition. J Forensic Sci. 1976;21(1):191-200. |
|  | Moorrees modification: London Atlas | 2010 | Atlas | 577 | X-ray | Panoramic X-ray | Atlas | "London Atlas" combining emergence data and the Moorress method, applicable from the first prenatal trimester to 23 years old. | AlQahtani SJ, Hector MP, Liversidge HM. Brief communication: The London atlas of human tooth development and eruption. Am J Phys Anthropol. 2010;142(3):481-490. |
|  | Nicodemo | 1974 | Atlas | 73 | X-ray | Panoramic X-ray | Foreign Language [Chinese] | Foreign Language [Chinese] | Nicodemo RA, Moraes LC, Médici Filho E. Tabela cronológica da mineralização dos dentes permanentes entre brasileiros. Rev Fac Odontol São José dos Campos. 1974;3(1):55-56. |
|  | Nolla | 1960 | Scoring | 1129 | X-ray | Full oral series | 70 stages | Each permanent tooth the left mandible is rated as per calcification stage (1-10) and values summed. | Nolla CM. The development of permanent teeth. 1952. |
|  | Schour & Massler | 1941 | Atlas | 340 | X-ray | Panoramic X-ray | 21 chronological ages | 21 stages identified: 2 prenatal followed by 0 months, 6mo , 9mo, 1 year, 16mo, 2y, 3y, 4y, 5y, 6y, 7y, 8y, 9y, 10y, 11y, 12y, 15y, 21y, 35y. | Massler M, Schour I, Poncher HG. Developmental pattern of the child as reflected in the calcification pattern of the teeth. Am J Dis Child. 1941;62(1):33-67. |
| Elbow | Sauvegrain | 1962 | Scoring | 53 | X-ray | Elbow: AP and Lateral | 27 points | Lateral condyle, trochlea, olecranon apophysis and proximal radial epiphysis evaluated. | Sauvegrain J, Nahum H, Bronstein H. Study of bone maturation of the elbow. In: Annales de Radiologie. Vol 5. ; 1962:542. |
|  | Sauvegrain modified: Dimeglio | 2005 | Scoring | 117 | X-ray | Elbow: AP and Lateral | 30 point | Intermediate scores added between 3 points, for a total of 30 points. | Accuracy of the Sauvegrain Method in Determining Skeletal Age During Puberty. 2005:1689-1696. |
|  | Sauvegrain modified: Dimeglio (Simplified) | 2001 | Scoring | 76 | X-ray | Elbow: Lateral only | 8 points | Simplified method in which only olecranon apophysis assessed. | Diméglio A. Growth in pediatrics orthopaedics. In: Lovell, Winter, eds. Vol. I chapter II, 5th ed. Lippincott; 2001 (in press). |
| Face | Braga | 2007 | Scoring | 58 | CT | CT | Linear Equation | 8 foramina identified, computer applies a cage/ mesh made and the shape is related to maturity via linear regression equation. | ﻿Braga J, Treil J. Estimation of pediatric skeletal age using geometric morphometrics and three-dimensional cranial size changes. Int J Legal Med. 2007;121(6):439-443. |
| Femur | Castriota-Scanderbe & De Micheli | 1995 | Scoring | 32 | Ultrasound | Hip Ultrasound | Continuous Scale | Femoral head cartilage thickness measured, decreases with increasing age and can be compared to table. | Castriota-Scanderbeg A, De Micheli V. Ultrasound of femoral head cartilage: a new method of assessing bone age. Skeletal Radiol. 1995;24(3):197-200. |
|  | Stull | 2014 | Scoring | 32 | X-ray | Femur: AP | Linear Equation | Based on femoral measurements, diaphyseal length can be correlated reliably with age until 6 years old, or with multivariate models entailing predominantly diaphyseal width for older individuals. | Lodox Statscan, invented in South Africa for mining industry . Simiilar to EOS. |
| Foot | Whitaker | 2002 | Scoring | 27 | X-ray | Foot AP & Lateral | 10 stages | Calcaneus primary and secondary ossification centers are rated 0-4, and on their fusion state from 0-4. All three scores are summed. | ﻿Whitaker JM, Rousseau L, Williams T, Rowan RA, Hartwig WC. Scoring system for estimating age in the foot skeleton. Am J Phys Anthropol Off Publ Am Assoc Phys Anthropol. 2002;118(4):385-392. |
|  | Whitaker | 2002 | Scoring | 27 | X-ray | Foot AP & Lateral | 10 stages | All distal phalangeal and proximal phalangeal primary and secondary ossification centers are rated 1-4, and on their fusion state from 1-4. All three scores are summed and converted to a 10 point scale. Uniquely, images were marked with a special score if ossification was not visible or obscured due to image quality or obstruction. | ﻿Whitaker JM, Rousseau L, Williams T, Rowan RA, Hartwig WC. Scoring system for estimating age in the foot skeleton. Am J Phys Anthropol Off Publ Am Assoc Phys Anthropol. 2002;118(4):385-392. |
| Hand-wrist | Björk & Helm | 1967 | Scoring | 563 | X-ray | Right Hand | 8 stages | Key events are recorded at the second finger, third finger, thumb sesamoid and radius are evaluated. | Björk A, Helm S. Prediction of the age of maximum puberal growth in body height. Angle Orthod. 1967;37(2):134-143. |
|  | Cameriere | 2006 | Scoring | 76 | X-ray | Left Hand | Linear Equation | The ratio of the area occupied by the carpal bones versus the total area of the radial and ulnar epiphysis and carpals is calculated and compared against a reference line. | Cameriere R, Ferrante L, Mirtella D, Cingolani M. Carpals and epiphyses of radius and ulna as age indicators. Int J Legal Med. 2006;120(3):143-146. |
|  | Cameriere modification: De Luca | 2016 | Scoring | 4 | X-ray | Left Hand | Linear Equation | Updated formula for greater accuracy. | De Luca S, Mangiulli T, Merelli V, et al. A new formula for assessing skeletal age in growing infants and children by measuring carpals and epiphyses of radio and ulna. J Forensic Leg Med. 2016;39(January):109-116. doi:10.1016/j.jflm.2016.01.030 |
|  | Chang et al. | 1990 | Scoring | 4 | X-ray | Hand (side not found) | 9 stages | "National Taiwan University Hospital Skeletal Maturity Index". Five locations on the thumb, index finger, middle finger and radius are evaluated. | Chang HF, Wu K-M, Chen KC. A cross-sectional study on the skeletal development of the hand and wrist from preadolescence to early adulthood among Chinese in Taiwan. Zhonghua ya yi xue hui za zhi. 1990;9(1):1-11. |
|  | Chinese National Sports Committee | 1992 | Foreign Language [Chinese] | 3 | X-ray | Foreign Language [Chinese] | Foreign Language [Chinese] | So-called 'Chinese Standard' method. Metacarpal, phalanges and carpal bone developmental stages are assessed. | ﻿Committee NS. Assessment of development of metacarpals, phalanges and carpals of Chinese people: national standard of People’s Republic of China. Beijing Natl Sport Comm. 1992. |
|  | Choi et al. | 2018 | Scoring | 5 | X-ray | Left or Right | Continuous | Area of the capitate and hamate is measured and summed. Value can be input to regression equation. | Choi JA, Kim YC, Min SJ, Khil EK. A simple method for bone age assessment: The capitohamate planimetry. Eur Radiol. 2018;28(6):2299-2307. |
|  | DeRoo &Schroder | 1976 | Atlas | 34 | X-ray | Hand (side not found) | Atlas | Similarities to Greulich-Pyle atlas, though initiated in Dutch European children. | de Roo T, Schröder HJ. Pocket Atlas of Skeletal Age. Springer Science & Business Media; 2012. |
|  | Luk | 2014 | Scoring | 22 | X-ray | Distal Radius-Ulna | 11 & 9 stages | "DRU" (distal radius ulna) method, in which the radius epiphysis is scored from 1-11 stages and the ulna from 1-9 stages, each can be individually compared to chronological age. | Luk KDK, Saw LB, Grozman S, Cheung KMC, Samartzis D. Assessment of skeletal maturity in scoliosis patients to determine clinical management: a new classification scheme using distal radius and ulna radiographs. Spine J. 2014;14(2):315-325. |
|  | Ebri | 1993 | Scoring | 1 | X-ray | Left Hand | Linear Equation | Maximum dimensions of carpal and phalangeal epiphyseal are measured and evaluated compared to a linear regression equation. | Ebri BT. New method for evaluating ossification of the carpal bone. From a study with 5225 Spanish children. Pediatrie. 1993;48(11):813-817. |
|  | Eklöf & Ringertz | 1967 | Scoring | 94 | X-ray | Left Hand | Linear Equation | Distances are measured that correspond to the width and length of the hand and wrist bones, in ten ossification centers. | Eklöf O, Ringertz H. A method for assessment of skeletal maturity. In: Annales de Radiologie. Vol 10. ; 1967:330-336. |
|  | Eklöf & Ringertz modification: 3 bone method | 2009 | Scoring | 1 | X-ray | Left Hand | Linear Equation | Abbreviated version for only 3 ossification centers. | Olivete CJ, Rodrigues ELL. ER5 and ER3: bone age assessment by simplifications of the Eklof and Ringertz method = Maturidade óssea: estimação por simplificações do método de Eklof e Ringertz. Rev Odonto Ciência. 2009;24(4):361-366. |
|  | Eklöf & Ringertz modification: 5 bone method | 2009 | Scoring | 1 | X-ray | Left Hand | Linear Equation | Abbreviated version for only 5 ossification centers. | Olivete CJ, Rodrigues ELL. ER5 and ER3: bone age assessment by simplifications of the Eklof and Ringertz method = Maturidade óssea: estimação por simplificações do método de Eklof e Ringertz. Rev Odonto Ciência. 2009;24(4):361-366. |
|  | Engström | 1983 | Scoring | 189 | X-ray | Left Hand | 5 stages | Only 4 key events at specific regions are noted: epiphysis of the 2nd proximal phalanx is wide as the diaphysis; the epiphysis of the 3rd middle phalanx caps its diaphysis; complete epiphyseal fusion of the distal phalanx of 3rd finger; complete union of the distal epiphysis of the radius. | ﻿Engström C, Engström H, Sagne S. Lower Third Molar Development in relation to Skeletal Maturity and Chronological Age. Angle Orthod. 1983;53(2):97-106. |
|  | Fishman | 1982 | Scoring | 664 | X-ray | Left Hand | 11 stages | Key events are recorded at the third finger, fifth finger, radius and thumb sesamoid appearance. Fishman described these as 'skeletal maturity indicators' for correlation with mandibular and maxillary development, therefore favoured by orthodontists. | Fishman LS. Radiographic evaluation of skeletal maturation: a clinically oriented method based on hand-wrist films. Angle Orthod. 1982;52(2):88-112. |
|  | Flory | 1936 | Atlas | 155 | X-ray | Right Hand | Atlas: newborn 19 years old | Phalangeal and carpal atlas. | Flory CD. Osseous development in the hand as an index of skeletal development. Monogr Soc Res Child Dev. 1936;1(3):i-141. |
|  | Gilsanz & Ratib | 2005 | Atlas | 216 | X-ray | Left Hand | Atlas | A digital hand atlas for assessment of phalanges, metacarpals and carpals. | Gilsanz V, Ratib O. Hand Bone Age: A Digital Atlas of Skeletal Maturity. Springer Science & Business Media; 2005. |
|  | Grave & Brown | 1976 | Scoring | 382 | X-ray | Hand (side not found/listed) | 14 stages | Fourteen ossification events are assessed first, second and third fingers in addition to the pisiform, hamate and distal radial epiphysis. Events are also correlated with peak growth velocity. | ﻿Grave KC, Brown T. Skeletal ossification and the a adolescent growth spurt. Am J Orthod. 1976;69(6):311-619. |
|  | Gretych | 2007 | Atlas | 117 | X-ray | Left Hand | Atlas | Second, third and fourth phalanges in addition to the carpals are evaluated with computer assistance in with reference to Caucasian, African-America, Asian and Hispanic children. | Gertych A, Zhang A, Sayre J, Pospiech-Kurkowska S, Huang HK. Bone Age Assessment of Children using a Digital Hand Atlas. Comput Med Imaging Graph. 2007;31(4-5):322-331. |
|  | Greulich & Pyle | 1950/ 1959 | Atlas | 7699 | X-ray | Left Hand | Atlas | The most well-known phalangeal and carpal atlas, first published in 1950, updated and expanded in 1959. | ﻿Greulich WW, Pyle SI. Radiographic Atlas Of Skeletal Development Of The Hand And Wrist. Am J Med Sci. 1959;238(3):393. |
|  | Greulich & Pyle modification: Individual Scoring | 1971 | Atlas | 53 | X-ray | Left Hand | Atlas | All bones are scored individually and then average bone age calculated. | Roche AF, Eyman SL, Davila GH. Skeletal age prediction. J Pediatr. 1971;78(6):997-1003. doi:10.1016/S0022-3476(71)80430-4. |
|  | Greulich & Pyle modification: Weighted Modification | 1972 | Atlas | 23 | X-ray | Right or Left Hand | Atlas | All bones are scored individually and summed by region (carpals, phalanges-metacarpals or forearm), and weighting is performed based on each region. | Kimura K. Skeletal maturation of children in Okinawa. Ann Hum Biol. 1976;3(2):149-155. |
|  | Greulich & Pyle modification: Weighted Modification | 1986 | Atlas | 4 | X-ray | Left Hand | Atlas | Regions are scored individually and summed according to a weighted formula. | Aicardi G, Di Battista E, Naselli A, Vignolo M, De Scrilli A. Affidabilitá dei piucomuni metodi di previsione della statura adulta in un campione di adolescenti italiani. Acta Med Auxol. 1986;18:55-65. |
|  | Greulich & Pyle modification: Weighted Modification | 1971 | Atlas | 10 | X-ray | Left Hand | Atlas | Higher weighting to metacarpals and phalanges II to V. | Peritz E, Sproul A. Some aspects of the analysis of hand‐wrist bone‐age readings. Am J Phys Anthropol. 1971;35(3):441-447. |
|  | Greulich & Pyle modification: Line Drawings | 1950 | Atlas | 90 | X-ray | Left Hand | Atlas | Greulich-Pyle Atlas converted to line drawings for easier use. | Buckler JMH, Buckler JMH. A Reference Manual of Growth and Development. Blackwell Science London; 1997. |
|  | Greulich & Pyle modification: Shorthand Bone Age | 2013 | Atlas | 30 | X-ray | Left Hand | Atlas | Abbreviated version of Greulich-Pyle atlas in which 10 specific events at the first and second digit, hamate, and radial epiphysis are assessed. | Heyworth BE, Osei DA, Fabricant PD, et al. The shorthand bone age assessment: a simpler alternative to current methods. J Pediatr Orthop. 2013;33(5):569-574. |
|  | Greulich & Pyle modification: Ultrasound | 2003 | Atlas | 56 | Ultrasound | Left Hand | Atlas | Tracings are made from ultrasound evaluations to represent the full hand and wrist, which can then be evaluated as per the GP atlas. | Bilgili Y, Hizel S, Kara SA, Sanli C, Erdal HH, Altinok D. Accuracy of skeletal age assessment in children from birth to 6 years of age with the ultrasonographic version of the Greulich-Pyle atlas. J Ultrasound Med. 2003;22(7):683-690. |
|  | Greulich & Pyle modification: 'Segmented’ | 2001 | Atlas | 51 | X-ray | Left Hand | Atlas | Seven regions are scored as groups - the carpals, metacarpals, proximal, medial and distal phalanges, radius, and ulna - and the sum divided by seven. | Mul D, Oostdijk W, Waelkens JJJ, Schulpen TWJ, Drop SLS. Gonadotrophin releasing hormone agonist treatment with or without recombinant human GH in adopted children with early puberty. Clin Endocrinol (Oxf). 2001;55(1):121-129. |
|  | Greulich & Pyle modification: Thumb Sesamoid only | 2008 | Scoring | 4 | X-ray | Left Hand | 2 stages | Binary staging of presence or absence of ulnar sesamoid of the first digit. Not recommended by the authors for use alone. | Chaumoître K, Adalian P, Colavolpe N, et al. Value of the sesamoid bone of the thumb in the determination of bone age. J Radiol. 2008;89(12):1921-1924. |
|  | Gu's method | - | - | - | - | - | - | Foreign Language [Chinese] | Zhang Z, Li K, Yu RJ, Zhang Q. Preliminary study on the applying value of two measurements for bone age in the cases of minors. Fa Yi Xue Za Zhi. 2004;20(4):212-214. |
|  | Haavikko | 1974 | - | 7 | - | - | - | Original article could not be obtained. | Haavikko K. Skeletal age estimated in a few selected ossification centres of the hand wrist. A simple method for clinical use. Proc Finn Dent Soc. 1974;70(1):7-14. |
|  | Hägg & Taranger | 1980 | Scoring | 325 | X-ray | Right Hand | 10 stages | First and third finger are assessed in addition to the radial distal epiphysis. First finger ulnar sesamoid (presence/absence), 3rd finger distal phalangeal fusion/non-fusion, 3rd finger middle phalangeal fusion progression; distal radial epiphyseal fusion progression. | ﻿Hägg U, Taranger J. Skeletal stages of the hand and wrist as indicators of the pubertal growth spurt. Acta Odontol Scand. 1980;38(3):187-200. |
|  | Hägg & Taranger modification: MP3 method | 1982 | Scoring | 536 | X-ray | Right Hand | 5 stages | "MP3" Method: Third middle phalanx only evaluated, based on distal epiphyseal plate morphology. | Hägg U, Taranger J. Maturation indicators and the pubertal growth spurt. Am J Orthod. 1982;82(4):299-309. |
|  | Hägg & Taranger modification: Modified MP3 Method | 2002 | Scoring | 76 | X-ray | Right Hand | 6 stage | "Modified MP3" Method: Third middle phalanx only evaluated, with 1 additional intermediate stage. | Rajagopal R, Kansal S. A comparison of modified MP3 stages and the cervical vertebrae as growth indicators. J Clin Orthod JCO. 2002;36(7):398. |
|  | Helm | 1971 | Scoring | 154 | X-ray | Right Hand | 7 stages | Only 7 key events at specific regions are noted. Sesamoid presence in the thumb, 2nd finger proximal phalanx, and distal, middle and proximal phalanges of the third finger are evaluated. | Helm S, Siersbaek-Nielsen S, Skieller V, Björk A. Skeletal maturation of the hand in relation to maximum puberal growth in body height. Tandlaegebladet. 1971;75(12):1223-1234. |
|  | Kopczyńska-Sikorska | 1969 | Atlas | 22 | X-ray | Left Hand | Atlas | Atlas of Polish children. | Kopczyńska-Sikorska J. Atlas Radiologiczny Rozwoju Kośćca Dłoni i Nadgarstka. Państwowy Zakład Wydawnictw Lekarskich; 1969. |
|  | Li Guozhen | - | - | - | - | - | - | Li Guozhens' "Percent Numeration" No description found, only references in Chinese language journals. | Zhang Z, Li K, Yu RJ, Zhang Q. Preliminary study on the applying value of two measurements for bone age in the cases of minors. Fa Yi Xue Za Zhi. 2004;20(4):212-214. |
|  | Liaokawa | - | - | - | - | - | - | No description found, only references in abstract of Chinese language journals. | |
|  | Mackay | 1952 | Scoring | 105 | X-ray | Left & Right Hand | 21 stages | Appearance of ossification centres of each carpal & phalanx in East Africans | ﻿Mackay DH, Service CM, Development C, Acts W. Skeletal maturation in the hand: a study of development in East Africa children. 1952. |
|  | Marti-Henneberg | 1974 | Scoring | 4 | X-ray | - | - | Original article could not be obtained. | Marti-Henneberg C, Patois E, Niiranen A, Roy MP, Masse NP. Bone maturation velocity. Compte Rendu la XIIe Reun des Équipes Charg des Études sur la Croissance le Développement l’Enfant Norm Paris, Cent Int l’Enfance, Paris. 1974:107-112. |
|  | Martins & Sakima | 1977 | Scoring | 46 | X-ray | Left Hand | 18 stages | Carpal ossification evaluated which can be compared to pubertal growth spurt. | Martins JCR, Sakima T. Considerações sobre a previsão do surto de crescimento puberal. Ortodontia. 1977;10(3):164-170. |
|  | Mentzel | 2005 | Scoring | 93 | Ultrasound | Right or Left Hand | Atlas-like result (score correlated to GP bone age) | BonAge' ultrasound device allows evaluation of the change in speed of waves across the distal radial and ulnar epiphyses which returns a skeletal age value correlated to the G-P atlas. | Mentzel H-J, Vilser C, Eulenstein M, et al. Assessment of skeletal age at the wrist in children with a new ultrasound device. Pediatr Radiol. 2005;35(4):429-433. |
|  | Modi | 1957/ 1969 | Atlas | 58 | X-ray | - | - | Original article could not be obtained. | Modi JP. Modi’s Textbook of Medical Jurisprudence and Toxicology. NM Tripathi; 1969. |
|  | Rachmiel | 2013 | Scoring | 3 | Ultrasound | Ultrasound | Linear | The distance across and speed of soundwave travel across the third phalanx, carpal region and wrist are measured. A linear equation gives output correlated to the GP atlas. | ﻿Rachmiel M, Naugolani L, Mazor-Aronovitch K, Levin A, Koren-Morag N, Bistritzer T. Bone age assessment by a novel quantitative ultrasound based device (SonicBone), is comparable to the conventional Greulich and Pyle method. Horm Res Pediatr. 2013;80(Suppl 1):35. |
|  | Roche | 1988 | Scoring | 379 | X-ray | Left Hand | Continuous Scale | Ninety-eight 'maturity indicators' across the radius, ulna, carpals, metacarpals and phalanges are recognised or measured, with different indicators assessed at different ages. Computer software returns a skeletal age with standard error and confidence limits. | Roche AF, Thissen D, Chumlea W. Assessing the Skeletal Maturity of the Hand-Wrist: Fels Method. Thomas; 1988. |
|  | Sempé & Pavia | 1979 | Scoring | 697 | X-ray | Left Hand | 1000 point scale | Assessment is converted to a 'Skeletal Maturity Level' corresponding to 0 at birth to 999 at full maturity. | ﻿Sempé M, Pédron G, Roy-Pernot M-P. Auxologie: Méthode et Séquences.; 1979. |
|  | Sempé & Pavia modification: Maturos 4.0 | 2001 | Atlas | 10 | X-ray | Left Hand | Atlas | Semi-automated method in which twenty-two maturity indicators are considered and the user selects between one of 3 software-suggested ratings for each region, depending on chronological age. | Bouchard M, Sempé M. “MATUROS 4.0” CD: un nouvel outil d’évaluation de la maturation squelettique. Biométrie Hum Anthropol. 2001;19(1-2):9-12. |
|  | Sato et al. | 1999 | Scoring | 11 | X-ray | Hand (side not found/listed) | Linear | Computer aided skeletal maturity system (CASMAS). Third phalanx is extracted automatically and epiphyseal, metaphyseal widths measured, in addition to the width of the overlapping regions, and a multiple regression equation reports the bone age. | ﻿Sato K, Ashizawa K, Anzo M, et al. Setting up an automated system for evaluation of bone age. Endocr J. 1999;46(Suppl):S97-S100. |
|  | Singer | 1980 | Scoring | 65 | X-ray | Hand (side not found) | 6 stages | All hand bones, with specific attention in the early stages to several events in the pisiform, hamate, ulna, second proximal phalanx, third middle and third distal phalanges. | Singer J. Physiologic Timing of Orthodontic Treatment. Angle Orthod. 1980;50(4):322-333. |
|  | Speyer | 1950 | Atlas | - | X-ray | Hand (side not found) | Atlas | Original article could not be obtained. | ﻿Speyer. Betekenis En Bepaling van de Skeletleeftijd. Leiden: Sijthoff; 1950. |
|  | Stuart | 1962 | Scoring | 68 | X-ray | Hand (side not found) | Ossification center appearance | Appearance and ossification of 29 centers in the hand wrist are evaluated. | Stuart HC, Pyle SI, Cornoni J, Reed RB. Onsets, completions and spans of ossification in the 29 bone-growth centers of the hand and wrist. Pediatrics. 1962;29(2):237-249. |
|  | Sugiura | 1961 | Scoring | 5 | X-ray | Hand (side not found) | - | Original article could not be obtained. | ﻿Sugiura Y, Nakazawa O, Kunishima Y, Aoki M, Ito H. A method of assessing skeletal age (2nd report). J Japanese Orthop Assoc. 1961;35:429-439. |
|  | Sugiura & Nakazawa | 1968 | Scoring | 4 | X-ray | Hand (side not found) | - | Scoring of hand and wrist centers, based on Japanese population. | Sugiura Y, Nakazawa O. Bone Age. Roentgen Diagnosis of Skeletal Development. Tokyo: Chugai-Igaku; 1968. |
|  | Tanner & Whitehouse: TW1 | 1959 | Scoring | 363 | X-ray | Left Hand | Continuous Scale | Carpal, metacarpal and phalangeal assessment. | ﻿Tanner JM. A new system for estimating skeletal maturity from the hand and wrist, with standards derived from a study of 2600 healthy British children. Part II Scoring Syst. 1959. |
|  | Tanner & Whitehouse: TW1 - B5 Modification | 2003 | Scoring | 6 | X-ray | Left Hand | Continuous Scale | Radius, ulna, capitate, trapezium and the proximal epiphysis of the first phalanx of the fifth finger are evaluated individually as per TW1 and summed. | Guimarey L, Morcillo AM, Orazi V, Lemos-Marini SH V. Validity of the use of a few hand-wrist bones for assessing bone age. J Pediatr Endocrinol Metab. 2003;16(4):541-544. |
|  | Tanner & Whitehouse: TW2 | 1972 | Scoring | 2786 (1975) | X-ray | Left Hand | Continuous Scale | Carpal, metacarpal and phalangeal assessment. Alternative methods included carpals only or radius-ulna-short (RUS) bone modifications. | ﻿Tanner JM, Whitehouse RH, Healy MJR, Goldstein H. A revised system for estimating skeletal maturity from hand and wrist radiographs with separate standards for carpals and other bones (TW II system). Stand Skelet Age. 1972. |
|  | Tanner & Whitehouse: TW2 MR: RUS | 2013 | Scoring | 47 | MR | Left Hand: Open Compact MR | Continuous Scale | TW2 radius-ulna-short bones method applied to MR images from an open compact MR. | ﻿Terada Y, Kono S, Tamada D, et al. Skeletal age assessment in children using an open compact MRI system. Magn Reson Med. 2013;69(6):1697-1702. |
|  | Tanner & Whitehouse: TW2 MR: Fully automated | 2014 | Scoring | 19 | MR | Left Hand MR | Continuous Scale | Fully automated method in which 3D MR images are are assessed by computer using the TW2 method. | ﻿Stern D, Ebner T, Bischof H, Grassegger S, Ehammer T, Urschler M. Fully automatic bone age estimation from left hand MR images. In: International Conference on Medical Image Computing and Computer-Assisted Intervention. Springer; 2014:220-227. |
|  | Tanner & Whitehouse: TW2 MR: RUS* | 2016 | Scoring | 14 | MR | Left Hand MR | Continuous Scale | T1 weighted 3D VIBE sequence used and hand-wrist evaluated as per TW2 - RUS. | Urschler M, Krauskopf A, Widek T, et al. Applicability of Greulich–Pyle and Tanner–Whitehouse grading methods to MRI when assessing hand bone age in forensic age estimation: A pilot study. Forensic Sci Int. 2016;266:281-288. |
|  | Tanner & Whitehouse: TW2 CASAS | 1994 | Scoring | 69 | X-ray | Left Hand MR | Continuous Scale | Computer Assisted Skeletal Assessment System (CASAS). User must position the radiograph under a camera, the computer then creates a digital representation which is scored automatically and can be output as per British or other populations. The process must be repeated individually for each bone. | Tanner JM, Gibbons RD. A computerized image analysis system for estimating Tanner-Whitehouse 2 bone age. Horm Res Paediatr. 1994;42(6):282-287. |
|  | Tanner & Whitehouse: TW3 | 2001 | Scoring | - | X-ray | Left Hand | Continuous Scale | Carpal, metacarpal and phalangeal assessment. Alternative methods included carpals only or radius-ulna-short bone modifications. | Tanner J, Healy M, Goldstein H, Cameron N. Assessment of Skeletal Maturity and Prediction of Adult Height (TW3 Method). 3rd ed. London: WB Saunders, Harcourt Publishers Ltd; 2001. |
|  | Tanner & Whitehouse: Sanders modification | 2008 | Scoring | 54 | X-ray | Left Hand | 8 stages | TW3 - RUS system scoring is applied to the second to fifth phalanges and metacarpals, in addition to the distal radius epiphysis. Stages were made with specific emphasis on their correlation with event in scoliosis curve progression. | Sanders JO. Maturity indicators in spinal deformity. J Bone Jt Surg - Ser A. 2008;89(SUPPL. 1):14-20. |
|  | Thiemann & Nitz | 1986 | Atlas | 93 (2006 edition) | X-ray | Hand (side not found) | Atlas | Atlas of German children. | ﻿Thiemann H, Nitz I. Roentgenatlas Der Normalen Hand Im Kindesalter. Leipzig: Thieme; 1986. |
|  | Thodberg | 2009 | Atlas/Scoring | 232 | X-ray | Left Hand | Atlas/Scoring | Automated method in which fifteen bones are identified by software and each is scored individually - first to fifth metacarpals, phalanges of the first, third and fifth digits, and the distal radial and ulnar epiphyses. Bone age can then be output as per Tanner-Whitehouse or GP Atlas values. | ﻿Thodberg HH, Kreiborg S, Juul A, Pedersen KD. The BoneXpert method for automated determination of skeletal maturity. IEEE Trans Med Imaging. 2009;28(1):52-66. |
|  | Todd | 1937 | Atlas | 335 | X-ray | Left Hand | Atlas | Atlas method. | Todd TW. Atlas of skeletal maturation. 1937. |
|  | Tomei | 2014 | Scoring | 10 | MR | Hand MR | 85 points | Radius, ulna, capitate, hamate, pisiform and first and third proximal phalanges and metacarpals are evaluated and scored. | Tomei E, Semelka RC, Nissman D. Text-Atlas of Skeletal Age Determination: MRI of the Hand and Wrist in Children. Wiley Online Library; 2014. |
|  | Wilkins | 1950 | - | 34 | - | - | Original article could not be obtained. | Method is not separated by genders. | ﻿Wilkins L. The diagnosis and treatment of endocrine disorders in childhood and adolescence. Charles C. Thomas, Springf. 1950;2. |
| Hip | Acheson | 1957 | Scoring | 245 | X-ray | Pelvic | 45 stages | "Oxford method": 9 regions of the hip are evaluated - iliac, triradiate cartilage, ischiopubic junction, pubis, ischium, acetabulum, femoral head, greater trochanter and lesser trochanter. | Acheson RM. The Oxford Method of Assessing Skeletal Maturity. Clin Orthop Relat Res. 1957;10:19-39. |
|  | Acheson modified: Modified Oxford | 1996 | Scoring | 97 | X-ray | Pelvic | 30 stages | Modified Oxford: only head of femur, greater trochanter, lesser trochanter, iliac crest and triradiate cartilage are evaluated. | Stasikelis PJ, Sullivan CM, Phillips WA, Polard AJ. Slipped capital femoral epiphysis: prediction of contralateral involvement. JBJS. 1996;78(8):1149-1155. |
|  | Triradiate cartilage | - | Scoring | - | X-ray | Pelvic | 2 stages | Open or closed stages | No original publication could be found. For a useful reference however see, Dimeglio A. Growth in Pediatric Orthopaedics. 2001:549-555. |
| Humerus | Li* | 2018 | Scoring | 4 | X-ray | Left Shoulder | 5 stages | Proximal humeral epiphyseal closure assessed from open plate until fusion. Peak height velocity correlation possible. | Li DT, Cui JJ, Devries S, et al. Humeral Head Ossification Predicts Peak Height Velocity Timing and Percentage of Growth Remaining in Children. J Pediatr Orthop. 2018;38(9):e546-e550. |
|  | Ogden | 1978 | Atlas | 47 | X-ray | Shoulder X-Ray | Atlas | Forensic specimen derived method, the proximal humerus ossification centers appearance, fusion with each other and the developing epiphysis are assessed. | Ogden JA, Conlogue GJ, Jensen P. Radiology of Postnatal Skeletal development: The proximal humerus. Skeletal Radiol. 1978;2(3):153-160. |
|  | Walker & Lovejoy | 1985 | Scoring | 137 | X-ray | Proximal humerus | 8 stages | Proximal humerus are evaluated by relative lucency. | Walker RA, Lovejoy CO. Radiographic changes in the clavicle and proximal femur and their use in the determination of skeletal age at death. Am J Phys Anthropol. 1985;68(1):67-78. |
| Iliac | Risser | 1958 | Scoring | 529 | X-ray | Pelvic | No stages | Full excursion of the iliac crest apophysis is correlated with the ending of spinal growth (no stages described at this time). | Risser JC. The Iliac apophysis; an invaluable sign in the manage- ment of scoliosis. Clin Orthop. 1958; 11: 111–119. |
|  | Risser: US system | - | Scoring | - | X-ray | Pelvic | 6 stages | Presence, extent of excursion divided into quarters and initiation of fusion of the iliac crest apophysis are evaluated. | First reference could not be found, as also noted by Bitan et al. For description of this method see: ﻿Bitan FD, Veliskakis KP, Campbell BC. Differences in the Risser grading systems in the United States and France. Clin Orthop Relat Res. 2005;(436):190-195. |
|  | Risser: EU system | - | Scoring | - | X-ray | Pelvic | 6 stages | Presence, extent of excursion divided into thirds, initiation and completion of fusion of the iliac crest apophysis are evaluated. | First reference could not be found, as also noted by Bitan et al. For description of this method see: ﻿Bitan FD, Veliskakis KP, Campbell BC. Differences in the Risser grading systems in the United States and France. Clin Orthop Relat Res. 2005;(436):190-195. |
|  | Risser: Combined | 1985 | Scoring | 146 | X-ray | Pelvic | 7 stages | Combined method in which stages 2-4 are altered and one intermediate stage "3/4" is added. | Stagnara P. Les Déformations Du Rachis: Scolioses, Cyphoses, Lordoses. Masson; 1985. |
|  | Risser modified: Triradiate evaluation | 2010 | Scoring | 55 | X-ray | Pelvic | 7 stages | Triradiate cartilage evaluation is included as an additional "-1" stage. | Nault M-L, Parent S, Phan P, Roy-Beaudry M, Labelle H, Rivard M. A modified Risser grading system predicts the curve acceleration phase of female adolescent idiopathic scoliosis. JBJS. 2010;92(5):1073-1081. |
|  | Risser modified: Risser 'plus' | 2015 | Scoring | 47 | X-ray | Pelvic | 8 stages | Triradiate cartilage is evaluated in addition to the iliac crest in a method combining EU and US systems. Recommended by SOSORT guidelines, 2015. | Negrini S, Hresko TM, O’Brien JP, et al. Recommendations for research studies on treatment of idiopathic scoliosis: Consensus 2014 between SOSORT and SRS non-operative management committee. Scoliosis. 2015;10(1):1-12. |
|  | Risser modified: Ultrasound | 1995 | Scoring | 41 | Ultrasound | Ultrasound | 6 stages | US Risser method applied by comparing presence of apophysis to the extent of iliac crest as palpated by clinician. | Wagner UA, Diedrich V, Schmitt O. Determination of skeletal maturity by ultrasound: a preliminary report. Skeletal Radiol. 1995;24(6):417-420. |
|  | Risser modified: Four stage method | 1985 | Scoring | 412 | X-ray | Pelvic | 4 stages | The extent of excursion is not scored, such that stages describe the absence of the apophysis, it's presence, ongoing fusion, or completed fusion. First described in forensic reports. | Webb PAO, Suchey JM. Epiphyseal union of the anterior iliac crest and medial clavicle in a modern multiracial sample of American males and females. Am J Phys Anthropol. 1985;68(4):457-466. |
|  | Schmidt | 2011 | Scoring | 57 | Ultrasound | Ultrasound Left Iliac Crest | 4 stages | The absence, presence, ongoing fusion and complete fusion of the left iliac crest apophysis are evaluated. | Schmidt S, Schmeling A, Zwiesigk P, Pfeiffer H, Schulz R. Sonographic evaluation of apophyseal ossification of the iliac crest in forensic age diagnostics in living individuals. Int J Legal Med. 2011;125(2):271-276. |
|  | Wittschieber | 2012 | Scoring | 35 | X-ray | Pelvic | 8 stages | Alternative method described in forensic evaluation, evaluating iliac crest apophyseal presence/absence, extent of excursion, extent of fusion, and completion of fusion. | Wittschieber D, Vieth V, Domnick C, Pfeiffer H, Schmeling A. The iliac crest in forensic age diagnostics: Evaluation of the apophyseal ossification in conventional radiography. Int J Legal Med. 2013;127(2):473-479. |
| Knee | Acheson | 1954 | Scoring | 215 | X-ray | Knee AP | 12 points | Femur and tibia are scored from 0-5 based on epiphyseal morphology (though not on the presence of fusion), patella and fibular epiphyses presence is scored 0 or 1. | Acheson RM. A method of assessing skeletal maturity from radiographs; a report from the Oxford child health survey. J Anat. 1954;88(4):498-508. |
|  | Dedouit | 2012 | Scoring | 91 | MR | Knee MR | 5 stages at 2 regions | Femur and tibial epiphyses are scored separately I-V and can be compared against age charts. | Dedouit, F., Auriol, J., Rousseau, H., Rougé, D., Crubézy, E. and Telmon, N., 2012. Age assessment by magnetic resonance imaging of the knee: a preliminary study. Forensic science international, 217(1-3), pp.232-e1. |
|  | Nakase | 2012 | Scoring | 24 | Ultrasound | Ultrasound Left Iliac Crest | 3 stages | Developing tibial tuberosity is described by 3 events adapted from Ehrenberg's radiographic description (1962) :increased cartilage, the presence of ossifying island, or connection by bone bridge to the tibial epiphyses. | Nakase J, Aiba T, Goshima K, et al. Relationship between the skeletal maturation of the distal attachment of the patellar tendon and physical features in preadolescent male football players. Knee Surgery, Sport Traumatol Arthrosc. 2014;22(1):195-199. |
|  | O'Connor | 2008 | Scoring | 70 | X-ray | Knee AP & Lateral | 15 stages | Epiphyses of the long bones around the knee are scored 0-4 and summed. Modified from McKern Stewart 1957 report from a forensic sample. | O’Connor JE, Bogue C, Spence LD, Last J. A method to establish the relationship between chronological age and stage of union from radiographic assessment of epiphyseal fusion at the knee: an Irish population study. J Anat. 2008;212(2):198-209. |
|  | Pennock* | 2018 | Atlas | 7 | MR | Knee MR | Atlas | Cross-sectional atlas using sagittal and coronal slices used to evaluate femur, tibia, fibula and patella based on ossification, shape and specific features of the regions such as tibial spine development and subchondral epiphyseal cartilage. | Pennock AT, Bomar JD, Manning JD, Diego S, Diego S. The creation and validation of a knee bone age atlas utilizing MRI. JBJS. 2018;100(4):e20. |
|  | Pyle & Hoerr | 1955 | Atlas | 140 | X-ray | Knee AP & Lateral | Atlas | Femur, tibia, fibula and patellar are assessed by atlas method. | Pyle SI, Hoerr NL. A Radiographic Standard of Reference for the Growing Knee. CC Thomas; 1955. |
|  | Roche-Wainer-Thissen | 1975 | Scoring | 117 | X-ray | Knee AP | 34 stages | Thirty four maturity indicators at the knee are evaluated. | Roche AF, Wainer H, Thissen D. Skeletal Maturity: The Knee Joint as a Biological Indicator. Plenum Medical Book Company; 1975. |
|  | Wang | 2010 | Scoring | 3 | X-ray | Knee AP & Lateral | - | Foreign Language [Chinese] | Wang YH, Zhu GY, Ying CL, Fan LH, Wan L. The trend of epiphyseal development of knee and ankle joints in teenagers and age estimation. Fa Yi Xue Za Zhi. 2010;26(2):91-96. |
| Lower limb | von Harnack | 1974 | - | - | - | - | - | Foreign Language [German] | von Harnack, G. A. "Determination of skeletal maturation in childhood (author's transl)." Zeitschrift fur Geburtshilfe und Perinatologie 178, no. 4 (1974): 237. |
| Mandible | Singer | 1987 | Scoring | 77 | X-ray | Lateral cephalogram | 2 stages | Antegonial notch depth is measured and rated whether "deep" (>3mm depth) or not. | Singer CP, Mamandras AH, Hunter WS. The depth of the mandibular antegonial notch as an indicator of mandibular growth potential. Am J Orthod Dentofac Orthop. 1987;91(2):117-124. |
| Metacarpal | Faruch-Bilfield | 2008 | Scoring | 4 | X-ray | Hand X-ray | Linear | Second metacarpal is measured to establish the ratio between epiphysial diameter and metaphysial diameter. This value can be correlated to chronological age. | Faruch-Bilfeld M, Dedouit F, Soumah M, et al. Value of radiographic evaluation of the second metacarpal in the determination of bone age. J Radiol. 2008;89(12):1930-1934. |
|  | Garn | 1972 | Scoring | 227 | X-ray | Left Hand | Linear | The longitudinal length of nineteen tubular bones of the hand are measured and compared against age standards. Prospective standard based on FELS population. | ﻿Garn SM, Hertzog KP, Poznanski AK, Nagy JM. Metacarpophalangeal length in the evaluation of skeletal malformation. Radiology. 1972;105(2):375-381. |
| Pubic symphysis | Omel'chenko & Sukhomlinova | 1976 | - | 1 | X-ray | Pelvic | - | Ischiopubic synchondrosis is evaluated. Foreign Language [Russian] | ﻿Omel’chenko RM, Sukhomlinova OP. Duration of synostosis of the ischiopubic synchondrosis. Arkh Anat Gistol Embriol. 1976;70(4):91-95. |
| Radius | Dvorak | 2007 | Scoring | 137 | MR | Coronal Left Hand MR | 6 stages | Distal radial growth plate is evaluated and initiation, extent or completion of fusion is graded I-VI. | ﻿Dvorak J, George J, Junge A, Hodler J. Age determination by magnetic resonance imaging of the wrist in adolescent male football players. Br J Sports Med. 2007;41(1):45-52. |
|  | Karami | 2014 | Scoring | 2 | Ultrasound | Ultrasound | Linear | The width of the hypoechoic growth plate is measured along the long radius' axis and compared to tables. Only cut-off points for the majority 16, 17 and 18 years old were investigated. | ﻿Karami M, Moshirfatemi A, Daneshvar P. Age determination using ultrasonography in young football players. Adv Biomed Res. 2014;3(1):174. |
|  | Schmidt | 2013 | Scoring | 38 | Ultrasound | Ultrasound | 4 stages | Distal radius evaluated for absence/presence of secondary center, the initiation of fusion, and the completion of fusion. (based on 4 stage ossification template as per Schulz et al, similar to many methods) | Schmidt S, Schiborr M, Pfeiffer H, Schmeling A, Schulz R. Age dependence of epiphyseal ossification of the distal radius in ultrasound diagnostics. Int J Legal Med. 2013;127(4):831-838. |
| Rib | Moskovitch | 2010 | Scoring | 25 | CT | Multi-slice CT | 5 stages | Sternal end of the right first rib is imaged and virtual reconstructions created, and assessed based on Kunos et al. macroscopic ratings, which yielded 5 stages around 15- 30 years old, based on shape, surface topography and form of the margin, and on the presence of osseous bridges in the sternocostal cartilage. | Moskovitch G, Dedouit F, Braga J, Rougé D, Rousseau H, Telmon N. Multislice computed tomography of the first rib: A useful technique for bone age Assessment. J Forensic Sci. 2010;55(4):865-870. |
|  | Michelson | 1934 | Scoring | 43 | X-ray | X-ray | 4 stages | Medial side of first rib is evaluated as first described in German by Ernst (1920), based on presence, extent and completion of epiphyseal ossification. | ﻿Michelson N. The calcification of the first costal cartilage among whites and negroes. Hum Biol. 1934;6(3):543. |
| Shoulder | Schaefer | 2015 | Scoring | 3 | X-ray | Shoulder X-Ray: AP, Axillary & Y-view. | 3 scores: 4 point, 3 point and 2 point | Proximal humerus , coracoid process and acromion are evaluated separately (on 4-, 3- and 2-point scales respectively). | Schaefer M, Aben G, Vogelsberg C. A demonstration of appearance and union times of three shoulder ossification centers in adolescent and post-adolescent children. J Forensic Radiol Imaging. 2015;3(1):49-56. |
| Sternum | Gemeler* | 2019 | Scoring | 3 | CT | Chest MDCT | 5 stages | Vertical fusion between three or four ossification centers of the sternal body and manubrium are assessed, and fusions proceeds superiorly with increasing maturity. (Measured in individuals up to 30 years old) | Gumeler E, Akpinar E, Ariyurek OM. MDCT evaluation of sternal development. Surg Radiol Anat. 2019;41(3):281-286. |
|  | Riach † | 1967 | Scoring | 23 | X-ray | Chest X-ray | Continuous | Tracings made of ossification center numbers and area calculated. Fusion is completed by 6-7 years old. (Method only described in forensic studies of excised sterna) | ﻿Riach IC. Ossification in the sternum as a means of assessing skeletal age. J Clin Pathol. 1967;20(4):589-590. |
| Wrist | Wang | 2014 | Scoring | 0 | X-ray | Left Hand | 5 stages | Five 'developmental stages' were applied for the radius and the ulna. No more information available - foreign Language [Chinese] | Wang YH, Wang ZS, Wei H, Wan L, Ying CL, Zhu GY. Automated assessment of developmental levels of epiphysis by support vector machine. Fa Yi Xue Za Zhi. 2014;30(6):422-426. |
|  | Kangne | 1999 | Scoring | 12 | X-ray | Hand (side not found/listed) | 4 stages | Distal radius epiphyseal fusion is evaluated from no fusion, partial fusion, near total fusion leaving only a thin line to complete fusion. | ﻿Kangne RN, Sami SA, Deshpande VL. Age estimation of adolescent girls by radiography. J Forensic Med Toxicol. 1999;16(1):20-26. |
|  | Kangne modification: Two-stage | 1999 | Scoring | 12 | X-ray | Hand (side not found/listed) | 2 stages | The first and second stages are combined, as are the third and fourth, resulting in 2 stages: 'not fused' and 'fused'. | ﻿Kangne RN, Sami SA, Deshpande VL. Age estimation of adolescent girls by radiography. J Forensic Med Toxicol. 1999;16(1):20-26. |
| Multi-region | Francis | 1940 | Scoring | 47 | X-ray | Many | Ossification center appearance | Ossification center appearances of 17 regions from humerus to iliac crest, sesamoids etc presented from 6-15 years old | Francis CC. The appearance of centers of ossification from 6 to 15 years. Am J Phys Anthropol. 1940;27(1):127-138. |
|  | Girdany & Golden | 1952 | Scoring | - | X-ray | Many | - | Based on the appearance of ossification centers, the wrist, elbow, shoulder, spine, hip, knee, ankle can be evaluated depending on age. | ﻿Girdany BR, Golden R. Centers of ossification of the skeleton. Am J Roentgenol Radium Ther Nucl Med. 1952;68(6):922-924. |
|  | Gök | 1985 | Atlas | 7 | X-ray | Many | Atlas | Turkish atlas of boys aged 11-22 based on the epiphyses of the shoulder, elbow, hand-wrist, and pelvic bones. | Gök Ş, Erölçer N, Özen C. Age Determination in Forensic Medicine. 2nd ed. Council of Forensic Medicine Press; 1985. |
|  | Graham | 1972 | - | 74 | - | - | - | Original article could not be obtained. | Graham CB. Assessment of bone maturation-methods and pitfalls. Radiol Clin North Am. 1972;10(2):185-202. |
|  | Schinz | 1939 | - | - | X-ray | Many | - | Hand, wrist, elbow, skull, hip, iliac crest, spine and knee are assessed. No further description found. Foreign Language [German] | Schinz H., Baensch WE, Friedl E, Uehlinger E. Lehrbuch der Röntgendiagnostik. In: Stuttgart: Thieme; 1950:761–776. |
|  | Spencer | 1981 | Scoring | 9 | Bone Scan | Full body scan with γ-camera. | Ossification center appearance | The appearance times of 71 ossification centers from 0-26 years old with 99m Tc-MDP or related compound uptake bone scans are listed. | Spencer RP, Sami S, Karimeddini M, Sziklas JJ, Rosenberg R. Role of bone scans in assessment of skeletal age. Int J Nucl Med Biol. 1981;8(1):33-38. |
|  | Zhu | 2008 | Scoring | 2 | X-ray | Many | not found foreign language | Seven regions are assessed and compared to so-called 'Grading Standards' :sternal end of clavical and left shoulder, elbow, carpal, hip, knee and ankle joints. No further description found in English (original article in Chinese). | Zhu GY, Fan LH, Zhang GZ, et al. Staging methods of skeletal growth by X-ray in teenagers. Fa Yi Xue Za Zhi. 2008;24(1):18-24. |
|  | Zhu: Knee-Ankle only | 2008 | Scoring | 3 | X-ray | Many | not found foreign language | Only knee and ankle assessed. No further description found in English (original article in Chinese). | Wang YH, Zhu GY, Ying CL, Fan LH, Wan L. The trend of epiphyseal development of knee and ankle joints in teenagers and age estimation. Fa Yi Xue Za Zhi. 2010;26(2):91-96. |
|  | Elgenmark: <5 years old | 1946 | Scoring | 110 | X-ray | Hemiskeleton | 24 age groups | Hand-wrist, knee, foot, prox femur, elbow, shoulder, applicable until 5 years old. In this ‘numerical’ method, the number of ossification centers is summed and this value is compared to chronological age. | Elgenmark O. The Normal Development of the Ossific Centres During Infancy and Childhood: A Clinical, Roentgenologic, and Statistical Study. Almqvist & Wiksell; 1946. |
|  | Ruckensteiner | 1931 | - | 47 | X-ray | - | Foreign Language [German] | Does not divide based on gender. | Ruckensteiner E. Die Normale Entwicklung Des Knochensystems Im Röntgenbild. Vol 15. Leipzig: Thieme; 1931. |
|  | Lurie | 1943 | Scoring | 18 | X-ray | Many | 20 stages | Hand-wrist, elbow, pelvis and foot on the right side. Age at appearance and fusion times are described, with specific landmarks highlighted as indicators of age such as the greater trochanter. | ﻿Lurie LA, Levy S, Lurie ML. Determination of bone age in children. J Pediatr. 1943;23(6):131-140. |
|  | Caffey | 1945 | Atlas | 1628 | X-ray | Many | - | A method made from several regions including hand-wrist as per Vickers & Vogt, it is not usuable for children older than 14 years old. Original article could not be located. | Caffey J, Silverman FN. Pediatric X-Ray Diagnosis. Chicago: Year Book Medical Publishers; 1945. |
|  | Vogt & Vickers | 1938 | Atlas | 52 | X-ray | Many | Atlas | Hand, wrist and feet from birth to 6 1/2 years old, ossification center appearance of the bones of the upper and lower extremities can be evaluated. Prospective atlas. | Vogt EC, Vickers VS. Osseous growth and development. Radiology. 1938;31(4):441-444. |
